# Phase-dependence of response curves to deep brain stimulation and their relationship: from essential tremor patient data to a Wilson–Cowan model

**DOI:** 10.1186/s13408-020-00081-0

**Published:** 2020-03-30

**Authors:** Benoit Duchet, Gihan Weerasinghe, Hayriye Cagnan, Peter Brown, Christian Bick, Rafal Bogacz

**Affiliations:** 1 Nuffield Department of Clinical Neuroscience, University of Oxford, Oxford, UK; 2 MRC Brain Network Dynamics Unit, University of Oxford, Oxford, UK; 3 Wellcome Centre for Human Neuroimaging, UCL Institute of Neurology, London, UK; 4 Oxford Centre for Industrial and Applied Mathematics, Mathematical Institute, University of Oxford, Oxford, UK; 5 Centre for Systems, Dynamics, and Control and Department of Mathematics, University of Exeter, Exeter, UK; 6 EPSRC Centre for Predictive Modelling in Healthcare, University of Exeter, Exeter, UK

**Keywords:** Deep brain stimulation, Essential tremor, Phase-locked stimulation, Phase response curve, Amplitude response curve, Wilson Cowan model, Focus model

## Abstract

Essential tremor manifests predominantly as a tremor of the upper limbs. One therapy option is high-frequency deep brain stimulation, which continuously delivers electrical stimulation to the ventral intermediate nucleus of the thalamus at about 130 Hz. Constant stimulation can lead to side effects, it is therefore desirable to find ways to stimulate less while maintaining clinical efficacy. One strategy, phase-locked deep brain stimulation, consists of stimulating according to the phase of the tremor. To advance methods to optimise deep brain stimulation while providing insights into tremor circuits, we ask the question: can the effects of phase-locked stimulation be accounted for by a canonical Wilson–Cowan model? We first analyse patient data, and identify in half of the datasets significant dependence of the effects of stimulation on the phase at which stimulation is provided. The full nonlinear Wilson–Cowan model is fitted to datasets identified as statistically significant, and we show that in each case the model can fit to the dynamics of patient tremor as well as to the phase response curve. The vast majority of top fits are stable foci. The model provides satisfactory prediction of how patient tremor will react to phase-locked stimulation by predicting patient amplitude response curves although they were not explicitly fitted. We also approximate response curves of the significant datasets by providing analytical results for the linearisation of a stable focus model, a simplification of the Wilson–Cowan model in the stable focus regime. We report that the nonlinear Wilson–Cowan model is able to describe response to stimulation more precisely than the linearisation.

## Introduction

Essential tremor (ET) is the most common movement disorder, affecting 0.9% of the population [1]. It predominantly manifests as a tremor of the upper limbs, and can severely affect daily-life. When medications are ineffective or not tolerated, thalamic deep brain stimulation (DBS) is a well-established therapy option. Clinically available DBS continuously delivers high-frequency (about 130 Hz) electrical stimulation to deep structures within the brain via an electrode connected to a pulse generator implanted in the chest. There is no agreement in the research community on the mechanisms of action of high-frequency DBS [2], but it is believed there is room for improvement in terms of efficacy, decrease in power usage, avoidance of habituation, and most importantly reduction of side effects [3]. Reported side effects of high-frequency thalamic DBS include speech impairment, gait disorders, and abnormal dermal sensations [4].

Because side effects are the main clinical bottleneck, improving high-frequency DBS generally means stimulating less by closing the loop on a signal related to motor symptoms, while maintaining clinical efficacy. One example of closed-loop DBS is adaptive DBS, whereby stimulation is triggered in Parkinson’s disease (PD) patients when pathological neural oscillation amplitude in the beta band is higher than a threshold. Compared to high-frequency DBS, it has been shown to improve motor performance, and reduce speech side effects in humans [5–7]. Another example is phase-dependent stimulation, which has been investigated in a computational model of PD [8], and in PD patients [9, 10].

Phase-locked DBS has recently been studied as a new therapy for ET [11]. Hand tremor is recorded, and the reduction in stimulation comes from stimulating with a burst of pulses according to the phase of tremor, only once per period of the tremor rather than continuously. In some patients, the strategy only requires half the energy delivered by high-frequency DBS for the same effect. Optimising phase-locked DBS requires a detailed understanding of the phase-dependence of the response across patients. However, data collection from phase-locked stimulation experiments has been restricted so far to small datasets because patients fatigue quickly. While direct analysis of the data has proven insightful [11], modelling phase-locked stimulation would allow predictions to be made from analytic and computational studies regarding the phase-dependence of the response to stimulation, and would open the door to supplement scarcely available patient data with synthetic data. The ability to easily generate large amounts of synthetic data could come in handy to help devise and test control algorithms. It could also be useful when trying to predict an effect that, because of noise in recordings, can only be deciphered when a large number of trials is available.

Tremulous hand movements are believed to be closely related to thalamic activity [12, 13], and it is believed that ET originates in the cerebellar–thalamic–cortical pathway [14]. However, detailed knowledge of how ET comes about is missing, which makes simple, canonical models natural candidates to study ET. Recently, phase-locked DBS was studied using Kuramoto phase oscillators which do not model interacting neural populations with distinct properties [15]. In the present work, we focus on a neural mass model, the Wilson–Cowan (WC) model, whose architecture can be mapped onto neural populations thought to be involved in the generation of ET, and allows for strong coupling between the populations. Additionally, stimulation can be delivered in the model to the most common stimulation site for ET, the ventral intermediate nucleus (VIM). The model describes the firing rates of an excitatory and an inhibitory population, and only has a few parameters, which makes it less prone to overfitting and significantly easier to constrain than more detailed models. The WC model has been shown to be adept at describing beta oscillations in PD [16, 17]. Moreover, the work presented in [18] provides evidence that the effects of high-frequency DBS for ET in a WC model are similar to the description given by conductance-based models. While the WC model has been used to design closed-loop strategies for PD [19, 20], whether a firing-rate model such as the WC can model the effects of phase-locked DBS has not been approached in the literature. Based on strong assumptions, Polina et al. reduced a WC model to a one-dimensional ordinary differential equation and looked at periodic forcing, but not in the context of DBS, and without attending to dependence on the phase of stimulation [21]. The present work will focus on reproducing the phase-dependent effects of phase-locked DBS measured in human data with a WC model.

Stimulation changes the phase and the amplitude of tremor and the dependence of these changes on the phase of stimulation can be quantified by the phase response curve (PRC, in this study change in tremor phase as a function of tremor phase) and the amplitude response curve (ARC, in this study change in tremor amplitude as a function of tremor phase). The ARC directly measures the change in tremor, hence the change in patient handicap, but both the ARC and the PRC are important to understand the effects of phase-locked DBS and potentially optimise the stimulation pattern. In mathematical neuroscience, PRCs and ARCs have been defined differently, mostly in the context of limit cycle models concerned with asymptotic response to infinitesimal perturbations; see for example [22–27]. In patients, DBS stimulation is not infinitesimal, and tremor data is very variable so stimulation happens in transient states. Therefore rather than considering an asymptotic description of the changes in phase and amplitude, we will be focussing on a close variant of the experimental response curve measurement methodology applied to blocks of stimulation in [11], which we will hereafter refer to as the “block method”. It provides a finite time response to a finite perturbation and relies on the changes in the Hilbert phase and amplitude of the tremor signal following blocks of phase-locked stimulation (more details in Sect. 2.1). The only exception to this will be in analytical derivations (Sect. 4), where a first order measurement of the response curves (i.e. measurement at the end of the stimulation period) will be used for tractability, as a simplified first approach to the model. For coherence with the experimental response curve measurement methodology, the notion of phase and amplitude used throughout will be the Hilbert phase and amplitude or approximately equivalent. It should also be noted that we are considering population response curves and not single neuron response curves. The vast majority of best performing WC models in reproducing patient data are found in this work to give rise to stable foci, where tremor dynamics is being reproduced by adding noise to the system, so we restrict our analytical considerations to stable foci.

Starting with the data, the narrative will be guided by the following questions. How do patient responses to phase-locked deep brain stimulation depend on phase? How do patient phase and amplitude response curves relate to one other? Can patient response curves and their relationship be described analytically in a simple linear model? Can we model patient tremor and better model response to phase-locked deep brain stimulation with a nonlinear WC model? The main contributions of this work are as follows. We first focus on the data and analyse patient response curves, identify a subset of datasets passing appropriate statistical tests, and characterise the relationship between PRC and ARC in these patients (Sect. 2). Following the introduction of our biologically motivated WC model (Sect. 3), we derive approximate analytical expressions that delineate the response to stimulation of a 2D dynamical system described by a linearised focus, with the goals of better understanding the constraints built in the model and of providing a first level of description of the data (Sect. 4). The derived response curves are close to sinusoidal, and a relationship between them is found, revealing similarities in shape and phase shift with patients who have statistically significant PRCs and ARCs. We then show that for these patients, the WC model can be fitted to the data and can reproduce the dependence of the effects of stimulation on the phase of stimulation. The model is fitted to the PRC and can reasonably predict the ARC, and notably what is approximately the best phase to stimulate (Sect. 5). We then proceed to compare the relationship between response curves in the linearised and the full model and conclude that nonlinearity is important to better reproduce the relationship found in patients (Sect. 6). Finally a discussion is provided (Sect. 7).

## Patient response curves and their phase relationship

In order to assess phase-dependence of the effects of DBS in patients, we extract PRCs and ARCs from patient’s tremor data, provide a statistical analysis of the response curves, and analyse their phase relationship when applicable. This data characterisation will inform our modelling approaches of the next sections, and we also introduce relevant concepts.

### Analysis method

**Figure 1:**
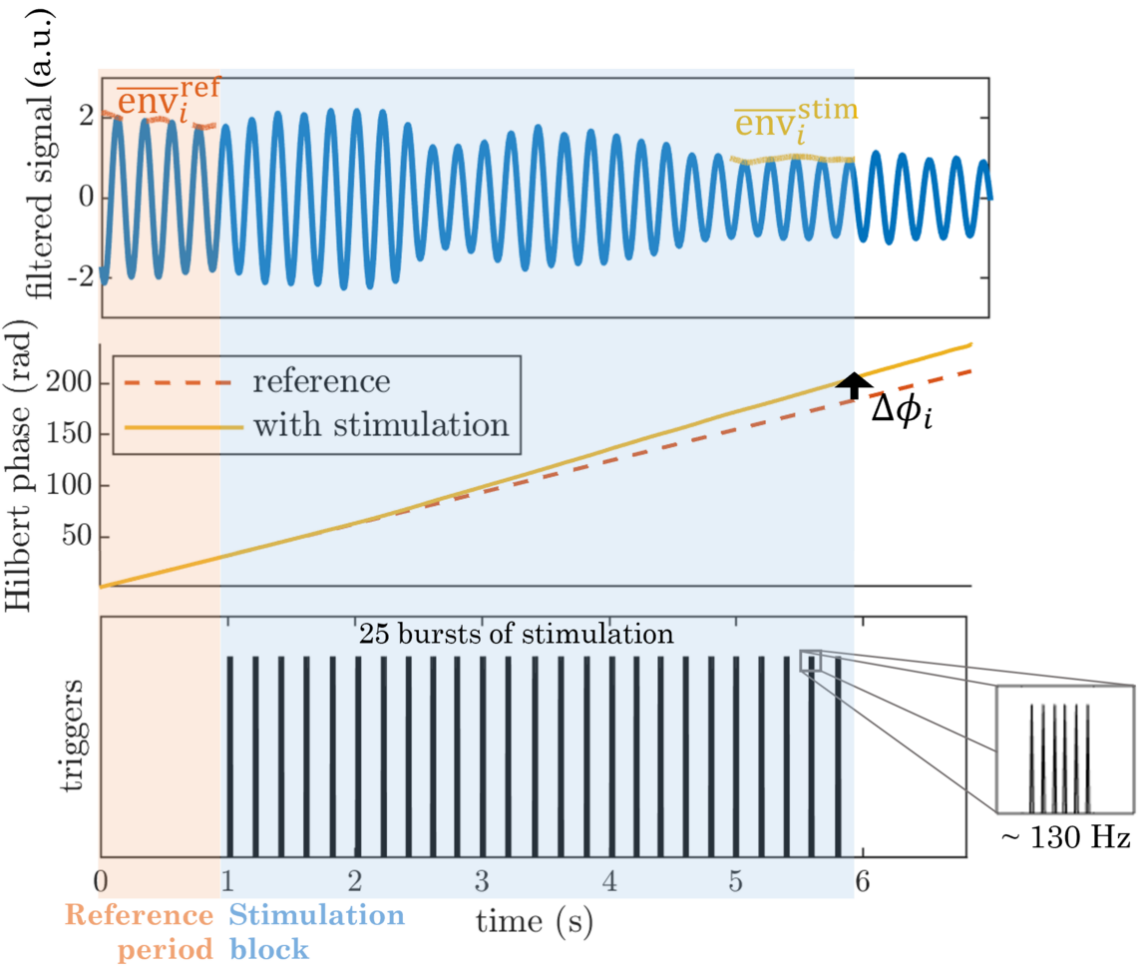
Example showing the block method applied to a block of stimulation with a target stimulation phase of 120 degrees. The three panels have the same horizontal axis. The reference period without stimulation before the block is highlighted in light orange, and the stimulation block itself in light blue. The filtered tremor is shown in blue in the upper panel. Stimulation triggers are shown in black in the lower panel. The 25 bursts of stimulation are each composed of a number of individual pulses at high frequency as shown in the zoomed-in insert. As shown in the middle panel, the change in phase $\Delta\phi_{i}$ due to the block of stimulation is obtained by comparing at the end of the block the actual Hilbert phase to a linear phase obtained by a straight line fit to the phase evolution 1 s before the block (reference period). The change in amplitude is given by the difference between the means $\overline{\text{env}}^{\text{stim}}_{i}$ and $\overline{\text{env}}^{\text{ref}}_{i}$ (top panel). Both the phase and amplitude responses are later normalised by the number of pulses in the block (not shown here)

We extract response curves from tremor acceleration data presented in [11]. The experimental paradigm in [11] is as follows. ET patients are fitted with an accelerometer to record their tremor acceleration, and DBS locked to the phase of tremor acceleration is provided in blocks of 5 s to the VIM of the thalamus, with 1 s without stimulation between blocks. An example of one such block of stimulation is shown in Fig. 1 in light blue, with the 1 s period without stimulation before the block highlighted in light orange (reference period). Each block targets a stimulation phase randomly selected out of 12 tremor phases (e.g. 120 degrees for the block shown in Fig. 1). Stimulation is delivered once per period at the target phase, in the form of a burst of four to six pulses at high frequency (130 Hz or higher). Details of the pulses making up a burst can be seen in the zoomed-in insert in Fig. 1. Tremor frequency being around 5 Hz and stimulation blocks lasting 5 s, there are about 25 bursts of stimulation at the same target phase per stimulation block. There are about 10 trials available per phase bin so about 120 stimulation blocks per patient (12 phase bins times around 10 trials per phase). The method described in [11] to obtain a patient’s response curves was specifically developed for this type of data, and we closely follow it and provide additional statistical analysis of the phase-dependence. We refer to our version of the method as the “block method” and denote the response curves obtained by bPRC and bARC, “b” standing for block. More specifically, we define the bPRC and the bARC according to the following procedure.

The dominant axis tremor acceleration recordings are bandpass-filtered (4 Hz band encompassing the patient tremor frequency content) and z-scored. The filter used is a Butterworth second order filter, which provides a maximally flat response in the passband [28]. Because this study focuses on phase, we perform zero-phase filtering by applying our filter in the forward and backward directions to avoid phase distortions. Since the resulting signal is narrow-band, the instantaneous phase $\phi(t)$ and amplitude $\mathrm{env}(t)$ are obtained as the Hilbert phase and amplitude (also called Hilbert envelope) of the processed tremor acceleration. The Hilbert phase and amplitude are given by the phase and the modulus of the analytic signal, respectively. The analytic signal is complex valued, and its real part is the signal (here processed tremor acceleration), while its imaginary part is the Hilbert transform of the signal. In short, we have $\operatorname{sig}(t) + \mathcal{H}(\operatorname{sig}(t)) = \mathrm{env}(t) e^{i \phi(t)}$, where $\operatorname{sig}(t)$ is the processed tremor acceleration and $\mathcal{H}$ denotes the Hilbert transform.

#### Obtaining the change in phase (bPRC)

For each block (we index blocks by the subscript *i*), a straight line $\widehat{\phi}^{ \mathrm{ref}}_{i}(t)$ is fitted to the evolution of the Hilbert phase $\phi_{i}(t)$ during the 1 s period without stimulation before the block (reference period; see middle panel in Fig. 1). The change in phase $\Delta\phi_{i}$ due to block *i* is given by the difference between the actual Hilbert phase at the end of the block and the phase of the fitted reference line evaluated at the end of the block (see middle panel in Fig. 1), i.e.

1 $$ \Delta\phi_{i} = \phi_{i}\bigl(t^{\mathrm{end}}_{i} \bigr) - \widehat{\phi}^{ \mathrm{ref}}_{i}\bigl(t^{\mathrm{end}}_{i} \bigr), $$

where $t^{\mathrm{end}}_{i}$ is the time of the end of block *i*. This phase response is divided by the number of pulses in blocks $n_{ \mathrm{pulses}}$ (on the basis of four pulses per burst for patient 4R and 4L, and six pulses per burst for the rest), which gives an average response for one pulse. The target phase at which stimulation is supposed to occur is known for each block, but phase tracking not being perfect, the actual Hilbert phase at which stimulation occurred is determined for each burst of stimulation as the circular mean of the Hilbert phase during the burst (unlike in the original study [11] where target phase is directly used). We take the circular mean of these burst angles for a given block as the actual mean phase of stimulation for the block, and denote it $\varPhi_{i}^{\mathrm{stim}}$ for block *i*. These values are then binned into 12 phases bins, and the change in phase is averaged within bins to obtain the bPRC. Put another way,

2 $$ \mathrm{bPRC}\bigl(\varPhi_{j}^{\mathrm{bin}}\bigr) = \frac{1}{n_{\mathrm{pulses}}n_{\mathrm{bin}_{j}}}\sum_{\varPhi_{i}^{ \mathrm{stim}}\in\mathrm{bin}_{j}} \Delta \phi_{i}, $$

where $\varPhi_{j}^{\mathrm{bin}}$ is the center phase of bin *j*, and $n_{\mathrm{bin}_{j}}$ is the number of blocks with $\varPhi_{i}^{\mathrm{stim}}$ falling in $\mathrm{bin}_{j}$.

#### Obtaining the change in amplitude (bARC)

For each block *i*, the change in amplitude $\Delta\mathrm{env}_{i}$ is given by the difference between the mean of the Hilbert amplitude during the last second of the block $\overline{\mathrm{env}}^{\mathrm{stim}}_{i}$ and the mean of the Hilbert amplitude during the one second without stimulation before the block $\overline{\mathrm{env}}^{\mathrm{ref}}_{i}$ (see top panel in Fig. 1):

3 $$ \Delta\mathrm{env}_{i} = \overline{\mathrm{env}}^{\mathrm{stim}}_{i} - \overline{\mathrm{env}}^{\mathrm{ref}}_{i}. $$

Similarly to the change in phase, this amplitude response is divided by the number of pulses in the block, and averaged across blocks in the same phase bin to obtain the bARC. Explicitly, we have

4 $$ \mathrm{bARC}\bigl(\varPhi_{j}^{\mathrm{bin}}\bigr) = \frac{1}{n_{\mathrm{pulses}}n_{\mathrm{bin}_{j}}}\sum_{\varPhi_{i}^{ \mathrm{stim}}\in\mathrm{bin}_{j}} \Delta \mathrm{env}_{i}. $$

#### Measuring response curves significance and PRC-ARC phase shift

In order to identify significant patient’s response curves, we performed two statistical analyses. First, bPRCs and bARCs were tested for a main effect of phase by means of a Kruskal–Wallis ANOVA (12 phase bins) to differentiate patients’ response curves that may be dominated by noise (which could be due to a lack of phase-dependent response or our inability to measure it, possibly because of an insufficient amount of data). Second, since we are expecting response curves to have a dominant first harmonic, the cosine model $y = c_{1} + \vert c_{2} \vert\cos(x+c_{3})$ was fitted to patients’ phase and amplitude response curves. We assessed via F-tests whether the cosine model was better at describing the data than a horizontal line at the mean ($y = c_{1}$, where $c_{1}$ is the mean change in phase or the mean change in amplitude). Including the less specific ANOVA test allows for more generality, as we do not wish to exclude patients with significant, but non-sinusoidal response curves. On the other hand, the cosine test is more likely to detect phase-dependent effects of stimulation in patients which indeed have sinusoidal response curves. We therefore define the following criterion for selection of a patient for further study in the rest of the manuscript.

#### Significance criterion

*Having both bPRC and bARC deemed significant under FDR control* (*see below*) *by at least one of the two tests—ANOVA test for a main effect of phase or cosine model F*-*test*.

In both cases, we address the multiple testing problem by controlling the false discovery rate (FDR) at 5%, which guarantees that the expectation of the number of false positives over the total number of positives is less than 5%. Because of the high number of rejections of the null hypotheses compared to the number of tests (5 out of 12 for the ANOVA, 6 out of 12 for the F-test; see Table 1), the total number of tests is a very poor estimator of the number of true null hypotheses, which is needed when controlling the FDR. Instead, we used a better estimator $\hat{m}_{0}$ of the number of true null hypothesis given by Story et al. [29], and applied an FDR control procedure based on this estimator (adaptive linear step-up procedure, reviewed in [30]).

**Table 1:** P-values of both statistical tests performed on patients’ response curves: Kruskal–Wallis ANOVAs testing a main effect for phase in patients’ response curves (third column), and cosine model F-tests (fourth column). P-values in bold are deemed significant with FDR control at the 5% level (separate FDR analyses per test type, $\hat{m_{0}} \approx8.42$ for the ANOVAs and $\hat{m_{0}} \approx7.37$ for the F-tests). Double stars indicate datasets satisfying our significance criterion as defined in Sect. 2.1

Patient	Type	ANOVA p-value	F-test p-value
1 **	bPRC	**0.0113**	**0.00993**
	bARC	0.1733	**0.0365**
3	bPRC	0.1097	0.448
	bARC	0.1591	0.500
4R	bPRC	0.3463	0.581
	bARC	0.2064	0.057
4L	bPRC	0.2895	0.352
	bARC	**0.0077**	0.200
5 **	bPRC	**4.925e−04**	**0.00906**
	bARC	**4.012e−06**	**0.00142**
6 **	bPRC	**4.815e−04**	**0.0122**
	bARC	0.0527	**0.0341**

Additionally, in datasets where both bPRC and bARC are significant according to the cosine F-test, the relationship between bPRC and bARC is quantified by the shift in phase between the cosine model fits to the bPRC and the bARC. In these datasets, the PRC-ARC shift between the bPRC and bARC is calculated as

5 $$ \phi_{\mathrm{PRC}}-\phi_{\mathrm{ARC}} \equiv c_{3}^{\mathrm{PRC}}-c_{3}^{\mathrm{ARC}} \pmod{2\pi}, $$

with $\phi_{\mathrm{PRC}}-\phi_{\mathrm{ARC}} \in [0,2\pi )$. Calculating a PRC-ARC shift in other cases is not meaningful. The PRC-ARC phase shift is an important quantity. Indeed, for PRCs and ARCs with a dominant first harmonic (close to sine curves), the ARC will be close to a scaled version of the PRC shifted in phase. The extent of the shift is given by the PRC-ARC phase shift. In other words, the minimum of the ARC (best phase to stimulate) will be at the minimum of the PRC plus the PRC-ARC shift. The shift highlights the difference in the phases of maximum sensitivity of the system in terms of its phase response and in terms of its amplitude response. As we will see later, the PRC-ARC shift will be a key differentiator between the nonlinear WC model and its linearisation in terms of their ability to describe the effects of phase-locked stimulation seen in data.

### Results of the analysis

**Figure 2:**
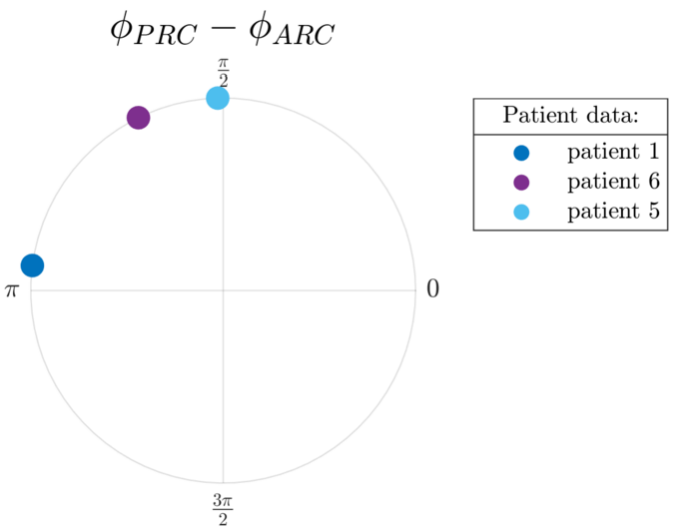
PRC-ARC shift in patients. Only showing patients with significant cosine model F-test for bPRC and bARC under FDR control. The calculated PRC-ARC shifts are in $[\frac{\pi}{2},\pi ]$

**Supplementary Figure 1:**
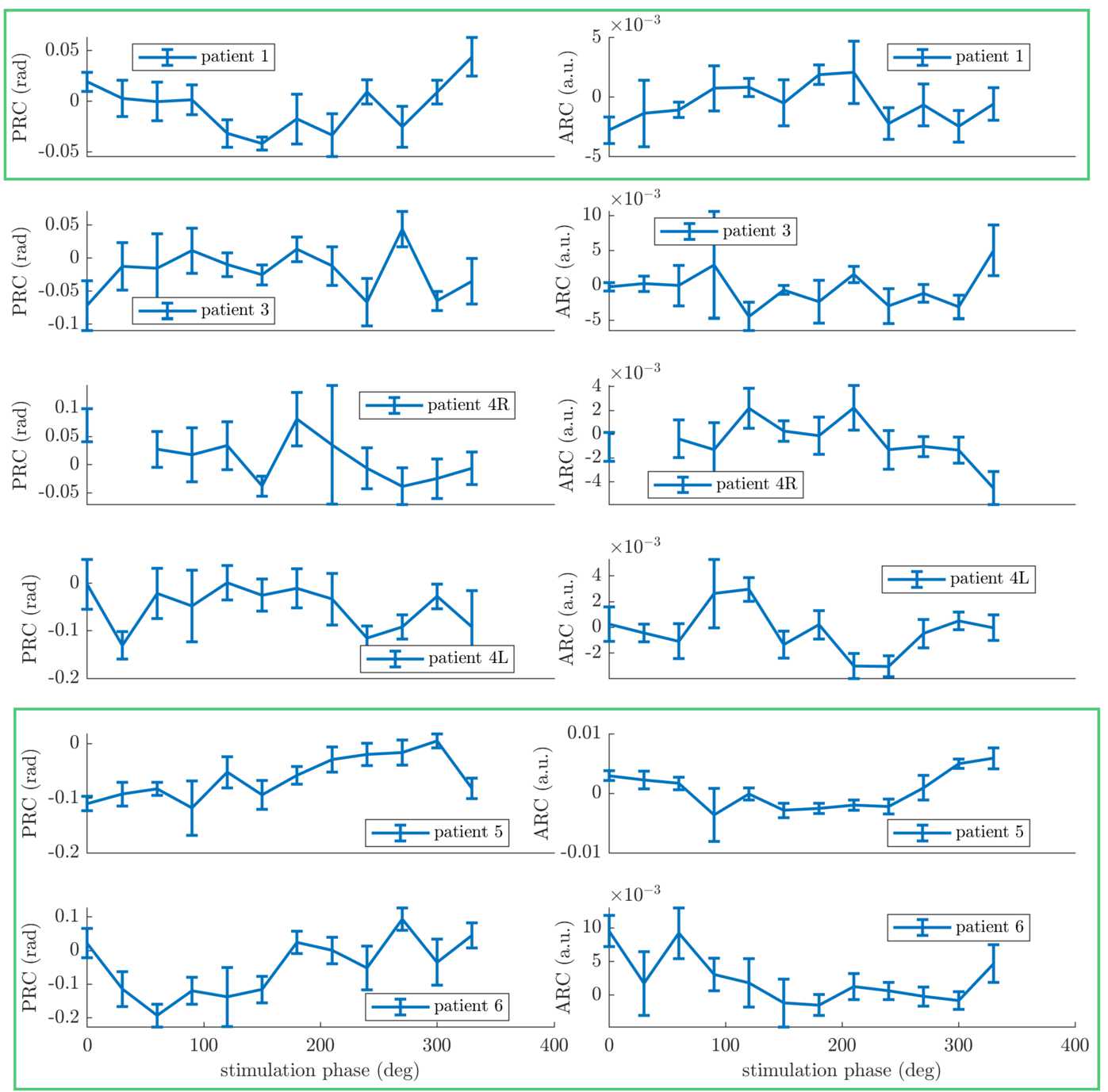
Patients’ bPRCs (first column) and bARCs (second column) obtained as described in Sect. 2.1. Datasets with both response curves significant according to at least one of our statistical tests under FDR control are highlighted with green rectangles

Analysing six datasets from the five patients included in [31] (datasets 4R and 4L are for the right and left upper limbs of the same patient) shows that half of the datasets satisfy our significance criterion. bPRCs and bARCs obtained are shown in Supplementary Fig. 1 in Appendix I, and results of the statistical tests are presented in Table 1. Based on the significance criterion defined in the previous section, patients 1, 5 and 6 are selected for further study, as both their bPRCs and their bARCs are found to be significant by the cosine F-test under FDR control. We note that patient 5 also has both his response curves deemed significant by the ANOVA test under FDR control. Datasets 3, 4R and 4L do not satisfy our selection criterion. In other words, for both tests, an effect of stimulation phase could not be found in at least one of their response curves (in most cases for both response curves, as seen in Table 1). In Fig. 2, the PRC-ARC shift $\phi_{\mathrm{PRC}}-\phi_{\mathrm{ARC}}$ is plotted for patients for whom the cosine model was deemed significant in describing both their bPRC and bARC (which happens to be the same subset as patients satisfying our significance criterion). Figure 2 shows that the PRC-ARC shift in significant datasets is in $[\frac{\pi}{2},\pi ]$, patients 5 and 6 being quite close to $\frac{\pi}{2}$.

## Implementation of the Wilson–Cowan model for essential tremor DBS

**Figure 3:**
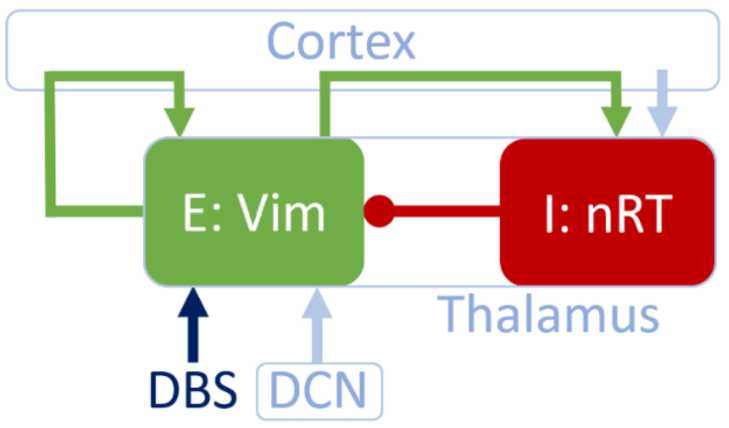
The WC model can describe the populations thought to be involved in the generation of ET. The excitatory population E and the inhibitory population I model, respectively, the VIM and the nRT of the thalamus. Arrows denote excitatory connections or inputs, whereas circles denote inhibitory connections. The VIM is the target of DBS and also receives an input from the deep cerebellar nuclei (DCN). The self-excitatory loop of the VIM, as well as the excitatory connection from VIM to nRT are via cortex

To model the experimental data described in the previous section, in particular the shape of the response curves and the PRC-ARC shift, we use a WC model that describes the interaction between an excitatory and an inhibitory population of neurons. Specifically, we map a two-population WC model without delays as described in [32] onto the anatomy of the thalamus (Fig. 3). The circuit we are about to describe is a good candidate, but not the only biologically plausible mapping of an excitatory/inhibitory loop in the context of tremor. In our candidate mapping, the VIM is modelled as an excitatory population, connected to an inhibitory population of the thalamus, the reticular nucleus (nRT). We model tremor by the activity of the excitatory population, and this is justified by the high coherence between ventral thalamic activity and electromyographic recordings of the contralateral wrist flexors [12, 13]. VIM and nRT are reciprocally connected (the excitatory projections from VIM to nRT are via Cortex). The VIM receives a constant input from the deep cerebellar nuclei (DCN) and is part of a self-excitatory loop via Cortex. nRT receives a constant cortical input. We add Gaussian white noise to this two-population WC, and the activity of the VIM, *E*, and the activity of the nRT, *I*, are described by the stochastic differential equations

6 $$ \textstyle\begin{cases} dE =F_{1}(E,I)\,dt + \zeta \,dW_{E}, \\ dI =F_{2}(E,I)\,dt + \zeta \,dW_{I}, \end{cases} $$

where $dW_{E}$ and $dW_{I}$ are Wiener processes, and *ζ* the noise standard deviation. We define

$$\begin{aligned} & F_{1}(E,I) =\frac{1}{\tau} \bigl(-E+f( \theta_{E}+w_{EE}E-w_{IE}I) \bigr), \\ & F_{2}(E,I) =\frac{1}{\tau} \bigl(-I+f( \theta_{I}+w_{EI}E) \bigr), \end{aligned}$$

with $w_{\mathrm{PR}}$ the weight of the projection from population “P” to population “R”, $\theta_{P}$ the constant input to population “P”, and *τ* a time constant (assumed to be the same for both populations). We use a sigmoid function,

$$\begin{aligned} & f(x) = \frac{1}{1+e^{-\beta(x-1)}}, \end{aligned}$$

parametrised by a steepness parameter *β* (same choice as in [32]). The VIM is the most common target of DBS for ET, which is why we model stimulation as a direct increase in *E*. Analytical expressions for response curves are out of reach for the full nonlinear model, which is why we study next a linearisation of a deterministic stable focus model to approximate the full model response and get a better understanding of the shape of its phase response curves and their relationship. This will provide a first level of description of the data.

## Response curves and their relationship in a focus model

This section aims to provide a basis for understanding how the effects of stimulation on phase and amplitude are coupled in the WC model, and for comparison with experimental data. We therefore derive approximate analytic expressions for the first order phase and amplitude responses to one pulse of stimulation in the linearisation of a 2D dynamical system that is described by a (stable) focus. Such a linearisation can be applied to the deterministic WC model given by Eq. (6) with $\zeta=0$ in the focus regime. We follow the previous section in modelling the tremor signal as the first coordinate of the dynamical system, and in providing stimulation pulses along the first dimension.

### Linearisation of a focus

To distinguish scalars and vectors more easily, vectors will be denoted in bold. Let $\dot{\mathbf {Z}}=F(\mathbf {Z})$ be a dynamical system, where $\mathbf {Z}\in\mathbb{R}^{2}$ and *F* is differentiable. The Jacobian of *F* is

7 $$ J = \begin{bmatrix} \frac{\partial F_{1}}{\partial Z_{1}} & \frac{\partial F_{1}}{\partial Z_{2}} \\ \frac{\partial F_{2}}{\partial Z_{1}} & \frac{\partial F_{2}}{\partial Z_{2}} \end{bmatrix} . $$

Let $\mathbf {Z^{*}}$ be a fixed point of *F*. If it is hyperbolic, the dynamics of $\mathbf {X}=\mathbf {Z}-\mathbf {Z^{*}}$ are approximated in the vicinity of the equilibrium $\mathbf {X} = \mathbf {0}$ by the linear equation

8 $$ \dot{\mathbf {X}} = J\bigl(\mathbf {Z^{*}}\bigr)\mathbf {X}, $$

where $J(\mathbf {Z^{*}})$ is the Jacobian evaluated at the fixed point. We will treat the case of Jacobians having complex conjugate eigenvalues $\lambda_{\pm}=\sigma\pm i\omega$. In particular, we are interested in stable hyperbolic foci, which imply $\sigma< 0$ and $\omega> 0$. The WC model can operate in that regime [32]. The nonhyperbolic case of the linearisation having purely imaginary complex conjugate eigenvalues will also be described for didactic purposes, although it is of little interest for patient fits. If $\mathbf {k}=\mathbf {a}+i\mathbf {b}$ is the right eigenvector associated with $\lambda_{+}$, and *K* and $K'$ coefficients determined according to initial conditions, the general real valued solution of (8) reads

9 $$ \mathbf {X}(t)= \bigl\{ K (\mathbf {a}\cos{\omega t}-\mathbf {b}\sin{\omega t} ) +K' (\mathbf {a}\sin{\omega t}+\mathbf {b}\cos{\omega t} ) \bigr\} e^{\sigma t}. $$

We will be using the following notations for the coordinates of the eigenvector:

10 $$ \mathbf {k} = \begin{bmatrix} a_{1} + i b_{1} \\a_{2} + i b_{2} \end{bmatrix} . $$

Equation (9) and what follows are not valid in the case of real eigenvalues, which are of no interest for our purposes (no rotation).

### Phase definition

The notion of phase is central to phase-locked stimulation, and in this section we define phase in a way that is approximately equivalent to the Hilbert phase, which is commonly used in the analysis of experimental data, and is used in the other sections of this manuscript. A typical signal only has one component, and the Hilbert transform provides a convenient way of reconstructing a phase from a single component. Despite being a protophase (see discussion section), the Hilbert phase is widely used to analyse experimental data (see for instance [9, 11, 33–35]), and this is the reason why we choose in our linearised system a phase definition approximately equivalent to it. We define a phase as $\phi=\omega t$ with a zero-phase point defined as the maximum of $X_{1}(t)$ (similarly to the Hilbert phase), which is therefore on the nullcline of the first coordinate. This phase definition is different from other common definitions such as the trajectory polar angle in the phase plane of a 2D system, or isochronal (asymptotic) phase. We demonstrate next that it is very close to the Hilbert phase of $X_{1}$ for slow decay compared to the rotation (this condition is verified in patient fits presented in Sect. 5.2; see Supplementary Table 2). It should be noted that this is generally only true for the linearisation. As the Hilbert phase is also the phase definition used in the other sections of this manuscript, the following proof ensures consistency.

We now establish equivalence of our phase definition with the Hilbert phase of $X_{1}$. Recall that we denote the Hilbert transform by $\mathcal{H}$. The Hilbert phase of $X_{1}$ is given by

11 $$ \phi^{\mathrm{Hilbert}}=\arctan{\frac{\mathcal{H}(X_{1}(t))}{X_{1}(t)}}. $$

A first step is to calculate the Hilbert transform of the signal $X_{1}(t)$. The Hilbert transform is a linear operator, and $X_{1}(t)$ is a linear combination of $s(t)s_{c}(t)$ and $s(t)s_{n}(t)$ with $s(t)=e^{\sigma\vert t \vert}$, $s_{c}(t)=\cos{\omega t}$, and $s_{n}(t) = \sin{\omega t}$ (see Eq. (9)). We show in Appendix A that the Hilbert transform $\mathcal{H}(s(t)s_{j}(t))$ can be approximated by $s(t) \mathcal{H}(s_{j}(t))$ for $j=c,n$. The Hilbert phase of $X_{1}$ is therefore given by

12 $$ \phi^{\mathrm{Hilbert}}=\arctan{\frac{\mathcal{H}(X_{1}(t))}{X_{1}(t)}} \approx\arctan{ \frac{\sqrt{\alpha^{2}+\beta^{2}} \sin (\omega t - \arctan {\frac{\alpha}{\beta}} )}{\sqrt{\alpha^{2}+\beta^{2}}\sin (\omega t + \frac{\pi}{2}- \arctan{\frac{\alpha}{\beta }} )}}, $$

where

$$\begin{aligned}& \alpha = K' a_{1} - K b_{1}, \\& \beta = K a_{1} + K' b_{1}. \end{aligned}$$

Using trigonometric identities, we obtain

13 $$ \phi^{\mathrm{Hilbert}} \approx\omega t - \arctan{ \frac{\alpha }{\beta}}. $$

In our setting, trajectories start at $t=0$ at the maximum of $X_{1}(t)$, and we have $\frac{\alpha}{\beta}=-\frac{\sigma}{\omega}$ (immediate with the coefficients of the reference trajectory $K_{\mathrm{ref}}$ and $K'_{\mathrm{ref}}$ introduced in Eq. (14) and given in Appendix B). Hence if $\omega\gg\vert\sigma\vert$, Eq. (13) yields $\phi^{\mathrm{Hilbert}} \approx\omega t$, which matches with our definition of phase *ϕ* (including our choice of zero-phase reference).

### Reference trajectory and stimulated trajectory

**Figure 4:**
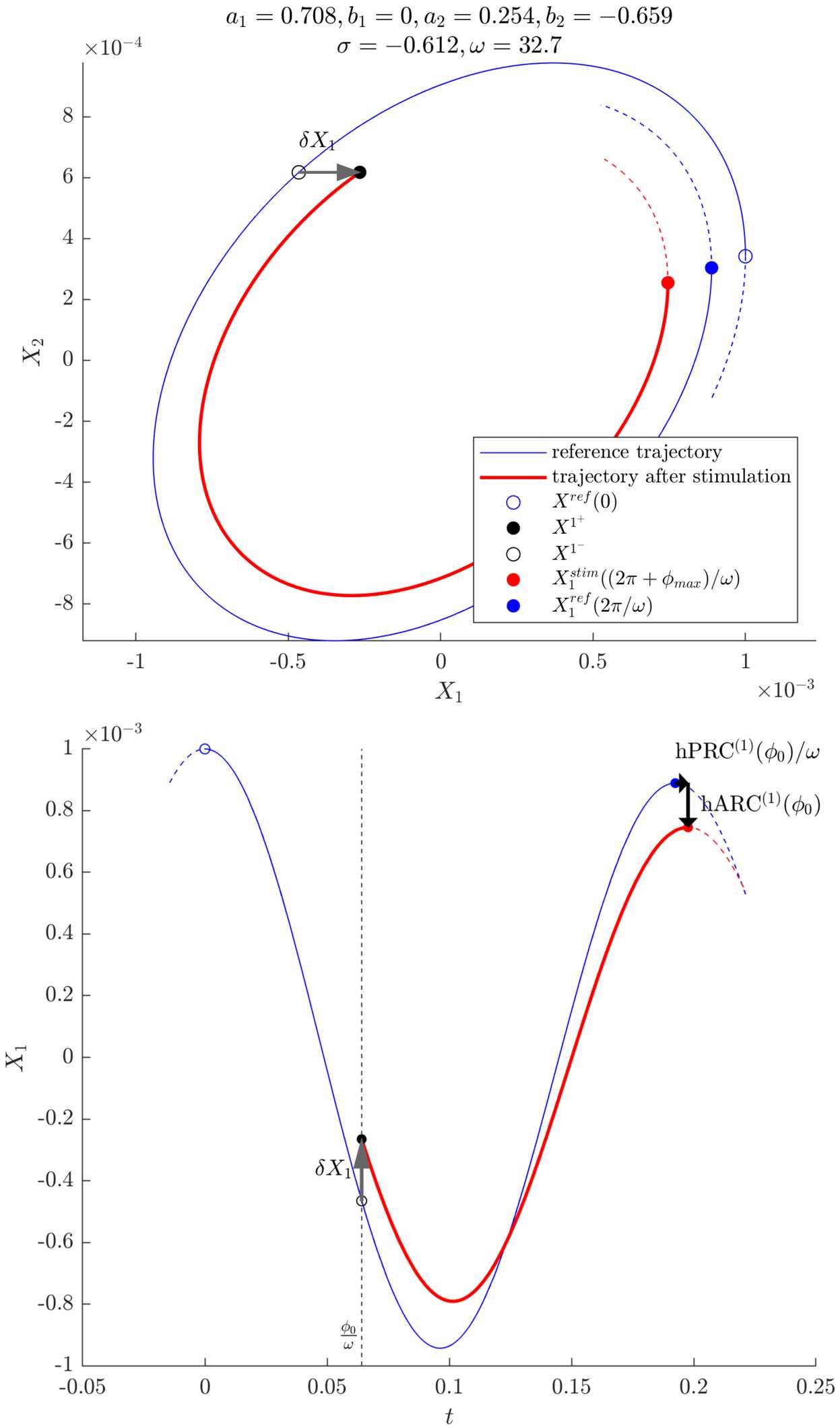
Illustration of the approach taken to derive expressions for the phase and amplitude responses in the linearisation of a 2D focus model. Top: phase plane, bottom: time series of $X_{1}$. The tremor is modelled by $X_{1}$, and the stimulation $\delta X_{1}$ is applied to $X_{1}$. The system shown corresponds to the linearised fit of patient 1 as described in Sect. 6.1

In order to calculate first order response curves for our phase definition, we will consider a reference trajectory without stimulation, and a trajectory that underwent an instantaneous stimulation pulse $\delta X_{1}$ at a stimulation phase $\phi_{0}$. The effects of stimulation on phase and amplitude will be measured at the next maximum of $X_{1}$ for both trajectories. We will denote these $\mathrm{hPRC}^{(1)}$ and $\mathrm{hARC}^{(1)}$ as they are first order responses based on a phase definition approximately equivalent to the Hilbert phase. A sketch of the method is provided in Fig. 4.

Expressions for the coefficients $K_{\mathrm{ref}}$ and $K'_{\mathrm{ref}}$ of the reference trajectory are derived in Appendix B. We want to study the effects of stimulating at phase $\phi_{0}$. The point of stimulation $\mathbf {X^{1^{-}}}$ at phase $\phi_{0}$ is expressed as

14 $$ \mathbf {X^{1^{-}}}= \bigl\{ K_{\mathrm{ref}} (\mathbf {a} \cos{\phi _{0}}-\mathbf {b} \sin{\phi_{0}} ) +K'_{\mathrm{ref}} (\mathbf {a}\sin{\phi_{0}}+ \mathbf {b} \cos{\phi_{0}} ) \bigr\} e^{\sigma \frac{\phi_{0}}{\omega}}. $$

An instantaneous stimulation $\delta X_{1}$ is applied at $\mathbf {X^{1^{-}}}$ as

15 $$ \mathbf {X^{1^{+}}}=\bigl(X_{1}^{1^{+}},X_{2}^{1^{+}} \bigr)=\bigl(X_{1}^{1^{-}}+\delta X_{1},X_{2}^{1^{-}} \bigr). $$

The trajectory after stimulation is still constrained by the dynamics given by Eq. (9), which allows for expressions for the coefficients on this new trajectory $K_{\mathrm{stim}}$ and $K'_{\mathrm{stim}}$ to be found (see Appendix C). To measure the change in phase and amplitude between the next peaks of the stimulated trajectory and the reference trajectory, the phase $\phi_{\mathrm{max}}$ of the next maximum of the first coordinate on the stimulated trajectory $X_{1}^{\mathrm{stim}}$ is needed (the phase of the next maximum of $X_{1}$ on the reference trajectory is 2*π*). A derivation for $\phi_{\mathrm{max}}$ is provided in Appendix D.

### Phase response

The first order phase response curve can be calculated based on the reference trajectory period $T_{0}$ and the stimulated trajectory period $T_{\mathrm{stim}}$, which is given by the sum of the time spent on the reference trajectory before stimulation and the time spent on the new trajectory after stimulation:

$$\begin{aligned}& T_{0} = \frac{2\pi}{\omega}, \\& T_{\mathrm{stim}} = \frac{(\phi_{0} - 0)+(2\pi+ \phi_{\mathrm{max}} - \phi_{0})}{\omega } = \frac{2\pi+ \phi_{\mathrm{max}}}{\omega}. \end{aligned}$$

For a phase response curve in radians, we obtain

16 $$ \mathrm{hPRC}^{(1)}(\phi_{0}) = 2 \pi\frac{T_{0} - T_{\mathrm {stim}}}{T_{0}} = - \phi_{\mathrm{max}}{(\delta X_{1})}, $$

where the phase $\phi_{\mathrm{max}}$ depends on the stimulation magnitude $\delta X_{1}$ (see Eq. (15)). The dependency enters through $K_{\mathrm{stim}}$ and $K'_{\mathrm{stim}}$ (see Eq. (41) in Appendix D and Eqs. (37) and (39) in Appendix C). A Taylor expansion around $\delta X_{1} = 0$ yields, to lowest order in $\delta X_{1}$ (for weak stimulation),

17 $$ \mathrm{hPRC}^{(1)}(\phi_{0}) \approx\frac{\delta X_{1}}{X_{1}^{0}} (A\cos{\phi_{0}}-B\sin{ \phi_{0}} ) C e^{-\sigma \frac{\phi_{0}}{\omega}} $$

with

$$\begin{aligned}& A = (a_{1} a_{2} + b_{1} b_{2})\omega-(a_{1} b_{2} - a_{2} b_{1}) \sigma, \\& B = (a_{1} b_{2} - a_{2} b_{1})\omega+(a_{1} a_{2} + b_{1} b_{2}) \sigma, \\& C = \frac{\omega}{(\omega^{2}+\sigma^{2})(a_{1} b_{2} - a_{2} b_{1})}. \end{aligned}$$

Although we are omitting the amplitude dependence in our notations for convenience in Eqs. (16) and (17), the first order PRC is found to be proportional to the inverse of the peak amplitude of the oscillations at the beginning of the stimulation period $X_{1}^{0}$. It is also directly proportional to the stimulation amplitude $\delta X_{1}$, and directly depends on phase via sinusoidal functions and a factor related to the decay. But unlike in the cosine test (Sect. 2), no assumption was made on a dominant first harmonic in our derivation. The constants *A*, *B*, and *C* only depend on the real and imaginary parts of the eigenvalue $\lambda_{+}$ (decay and rotation) and the associated eigenvector **k**.

### Amplitude response

For our purposes we are interested in the amplitude of the first coordinate, and the first order ARC is obtained as the difference in first coordinates between the stimulated and the reference trajectories evaluated at their respective next peak after stimulation. It should be noted this is approximately equivalent to a first order change in Hilbert amplitude, at least for $\omega\gg\vert\sigma\vert$. The first order ARC is calculated as

18 $$ \mathrm{hARC}^{(1)}(\phi_{0}) = X_{1}^{\mathrm{stim}} \biggl( \frac{2\pi+\phi_{\mathrm{max}}{(\delta X_{1})}}{\omega} \biggr)-X_{1}^{\mathrm{ref}} \biggl( \frac{2\pi}{\omega} \biggr). $$

A Taylor expansion around 0 yields, to lowest order in $\delta X_{1}$,

19 $$ \mathrm{hARC}^{(1)}(\phi_{0}) { \approx} \delta X_{1} (\cos{\phi_{0}}+D \sin{ \phi_{0}} ) e^{-\sigma\frac{\phi_{0}-2\pi}{\omega}} $$

with

$$ D = \frac{a_{1} a_{2} + b_{1} b_{2}}{a_{1} b_{2} - a_{2} b_{1}}. $$

Interestingly, the first order ARC close to the fixed point does not depend on the amplitude of the oscillations $X_{1}^{0}$. As expected, the first order ARC is directly proportional to the stimulation amplitude $\delta X_{1}$. Similarly to the first order PRC, it directly depends on phase via sinusoidal functions and a factor related to the decay, and the constant *D* only depends on **k**. The obvious similarities between the first order PRC and ARC suggest there may be a relationship between the two.

### Relationship between first order PRC and ARC

We seek a relationship involving the derivative of the first order PRC, which, based on Eq. (17), is given by

20 $$ -\frac{d\mathrm{hPRC}^{(1)}(\phi_{0})}{d\phi_{0}} \approx \frac{\delta X_{1}}{F X_{1}^{0}} ( \cos{ \phi_{0}} + G\sin{\phi_{0}}) e^{-\sigma\frac{\phi_{0}-2\pi}{\omega}} $$

with

$$\begin{aligned}& F = \frac{(a_{1} b_{2} - a_{2} b_{1}) (\omega^{2} +\sigma^{2} )}{ (a_{1} b_{2} - a_{2} b_{1}) (\omega^{2} -\sigma^{2} ) +2 (a_{1} a_{2} +b_{1} b_{2} )\omega\sigma} e^{\frac{2\pi\sigma}{\omega}}, \\& G = \frac{(a_{1} a_{2} + b_{1} b_{2})(\omega^{2}-\sigma^{2})-2(a_{1} b_{2} - a_{2} b_{1})\omega\sigma}{(a_{1} b_{2} - a_{2} b_{1})(\omega ^{2}-\sigma^{2})+2(a_{1} a_{2} + b_{1} b_{2})\omega\sigma}. \end{aligned}$$

For $\omega\gg\vert\sigma\vert$, we have

$$\begin{aligned}& F = \frac{(a_{1} b_{2} - a_{2} b_{1}) (1+ (\frac{\sigma}{\omega } )^{2} )}{ (a_{1} b_{2} - a_{2} b_{1}) (1- (\frac{\sigma}{\omega} )^{2} ) +2 (a_{1} a_{2} +b_{1} b_{2} )\frac{\sigma}{\omega}} e^{\frac{2\pi\sigma}{\omega}} \approx1 - 2(D-\pi) \frac{\sigma}{\omega} \approx1, \\& G = \frac{(a_{1} a_{2} + b_{1} b_{2}) (1- (\frac{\sigma}{\omega } )^{2} )-2(a_{1} b_{2} - a_{2} b_{1}) (\frac{\sigma }{\omega} )}{(a_{1} b_{2} - a_{2} b_{1}) (1- (\frac {\sigma}{\omega} )^{2} )+2(a_{1} a_{2} + b_{1} b_{2}) (\frac{\sigma}{\omega} )} \approx D - 2\bigl(1+D^{2}\bigr) \frac{\sigma}{\omega} \approx D. \end{aligned}$$

Therefore in that case the first order ARC is approximately the opposite of the derivative of the first order PRC scaled by the peak amplitude at the beginning of the stimulation period (in general, the scaling factor is $FX_{1}^{0}$):

21 $$ - X_{1}^{0}\frac{d\mathrm{hPRC}^{(1)}(\phi_{0})}{d\phi_{0}} \approx \mathrm{hARC}^{(1)}(\phi_{0}). $$

For a slow decay compared to the rotation, the PRC-ARC shift in the linearisation of a focus will therefore be close to $\frac{\pi}{2}$, which is the value observed for patient 5 (see Fig. 2). A detailed analysis of the PRC-ARC shift in the model is provided in Sect. 6.

### Applications to simple systems

We turn to simple examples of linear systems to illustrate the results of the previous sections, in particular how the strength of the decay affects the sinusoidal character of the response curves and the PRC-ARC shift, and how a tilted ellipsoid flow impacts the response curves. Additionally, links to the WC model are provided when possible. In what follows, response curves are given for $\delta X_{1} = 2 \times10^{-4}$ and $X_{1}^{0} = 10^{-3}$, $X_{1}^{0}$ being a maximum of $X_{1}$ as a function of time (these only act as scaling factors of the response curves and will not change their shape).

#### Circular flow without decay

As an introductory example, let us consider a simple circular flow for which the *J* matrix is

$$ J_{\mathrm{circ}} = \begin{bmatrix} 0 & -1 \\ 1 & 0 \end{bmatrix} . $$

**Figure 5:**
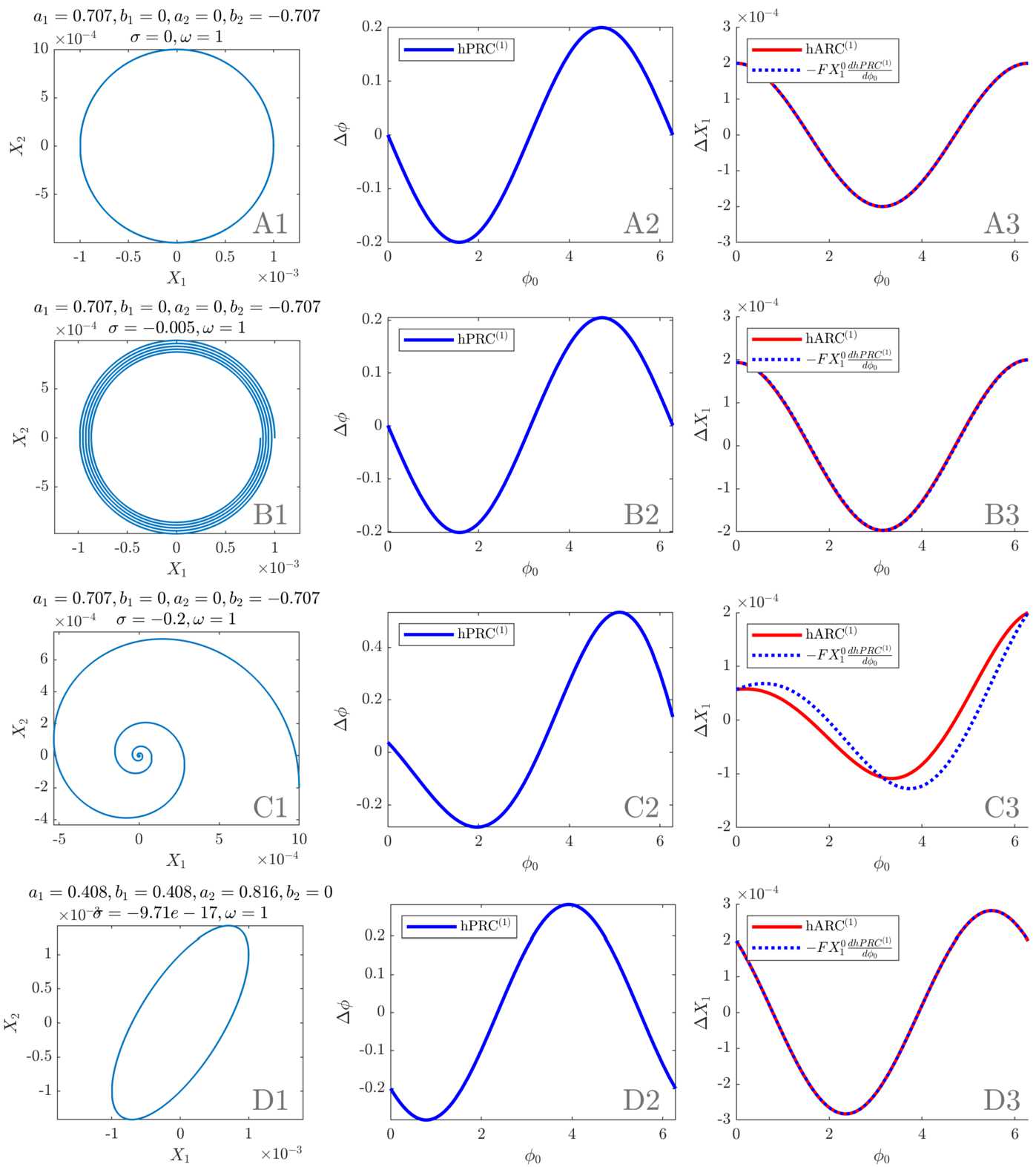
Analytical results in simple systems (initial conditions as in the main text). First column: phase space. Second column: first order PRC as per Eq. (17) (scaling valid for the first cycle). Third column: first order ARC as per Eq. (19) and opposite of the derivative of the first order PRC scaled by $FX_{1}^{0}$. Panel **A** corresponds to $J_{\text{circ}}$ (circular flow, no decay), panel **B** to $J_{\text{circ}}^{\text{slow}}$ (circular flow, slow decay), panel **C** to $J_{\text{circ}}^{\text{fast}}$ (circular flow, fast decay), and panel **D** to $J_{\text{ellip}}$ (tilted elliptic flow, no decay)

The eigenvalues of $J_{\mathrm{circ}}$ are ±*i* so the results of the previous sections can be applied. Equations (17) and (19) are plotted for this system with our choice of $\delta X_{1}$ and $X_{1}^{0}$. The result for the first order PRC is shown in Fig. 5, panel A2, and for the first order ARC in panel A3. For this system, $\sigma= 0$, and $\mathrm{hPRC}^{(1)}$ is simply the opposite of a sine, $\mathrm{hARC}^{(1)}$ simply a cosine. Moreover, $G = D$ (see Sect. 4.6) and Eq. (21) is exact, as exemplified in Fig. 5, panel A3. The amplitude response curve $\mathrm{hARC}^{(1)}$ is obtained by only scaling the derivative of $\mathrm{hPRC}^{(1)}$ by $-X_{1}^{0}$ as $a_{2} = b_{1} = 0$ and $F = 1$. Note that WC parameters for which the system’s Jacobian at the fixed point is $J_{\mathrm{circ}}$ cannot be found as the second diagonal term cannot be 0, at least in the version of the WC model used in this work (see equation (44) in Appendix E).

#### Circular flow with decay

We can introduce a slow decay (Fig. 5, panel B) and then a fast decay (Fig. 5, panel C) in the circular flow. We choose the *J* matrices

$$ J_{\mathrm{circ}}^{\mathrm{slow}} = \begin{bmatrix} -5{\times}10^{-3} & -1 \\ 1 & -5{\times}10^{-3} \end{bmatrix} , \qquad J_{\mathrm{circ}}^{\mathrm{fast}} = \begin{bmatrix} -2{\times}10^{-1} & -1 \\ 1 & -2{\times}10^{-1} \end{bmatrix} . $$

The slow decay leads to a scaling factor $F \approx1$, and the approximation of Eq. (21) is very good, as $\omega\gg\vert\sigma\vert$ (see Fig. 5, panel B3, close match of the ARC and the scaled derivative of the PRC, hence a shift close to $\frac{\pi}{2}$). The case of the fast decay corresponds to $\omega= 5 \vert\sigma\vert$. The first order PRC and ARC no longer look like pure sinusoids and the approximation relating the response curves is less accurate ($\omega= 200 \vert\sigma\vert$; see Fig. 5, panel C3), highlighting a shift different from $\frac{\pi}{2}$. It is also more obvious that the first order response curves are not periodic due to the measurement of the changes in phase and amplitude at the end of the stimulation period. It is possible to find WC parameters for which the system’s Jacobian at the fixed point is $J_{\mathrm{circ}}^{\mathrm{slow}}$ or $J_{\mathrm{circ}}^{\mathrm {fast}}$. How such parameters are found is explained in Appendix E, and the results are presented in Supplementary Table 1 in Appendix J. In both cases, $w_{IE} = w_{IE}$, and $w_{EE}=0$.

#### Tilted elliptic flow without decay

The tilted elliptic flow without decay of Fig. 5, panel D, corresponds to the *J* matrix

$$ J_{\mathrm{ellip}} = \begin{bmatrix} 1 & -1 \\ 2 & -1 \end{bmatrix} . $$

The first order PRC and ARC are sums of a sine and a cosine, which brings a horizontal shift in phase for both curves compared to a circular flow without decay. The eigenvalues are still purely imaginary, but *F* is no longer one. Because $\sigma= 0$, the relationship of Eq. (21) is still exact (see Fig. 5, panel D3). It is possible to find WC parameters for which the system’s Jacobian at the fixed point is $J_{\mathrm{ellip}}$ (see Supplementary Table 1 in Appendix J). Patient fits fall in the category of (potentially tilted) elliptic flows with decay, and will be dealt with in Sect. 6.1.

For a slow decay compared to the rotation, the linearised stable focus model exhibits close to sinusoidal response curves and a PRC-ARC shift close to $\frac{\pi}{2}$ as shown by Eq. (21). This is verified in Fig. 5, as the scaled first order PRC very closely match the ARC (except in panel C where the decay is fast). When contrasted with patient data (response curves passing the cosine model F test and PRC-ARC shifts in $[\frac{\pi}{2},\pi ]$ as shown in Fig. 2), these results already provide a good level of description of the data, but also a strong motivation to fit the more complex nonlinear WC model to data.

## Fitting the full Wilson–Cowan model to patient data and response to phase-locked stimulation

### Fitting procedure

With the insights on the linearised stable focus response curves given by the previous section in mind, and to provide a more accurate level of description of the data in particular in terms of PRC-ARC shift, we now turn to fitting our stochastic neural mass model introduced in Sect. 3 (Eq. (6)) to patient data. The model is fitted to features (also known as summary statistics) extracted from patient tremor recordings. The parameters we fit are shown in Table 2, and include model parameters, stimulation magnitude, and stimulation delay (time between when the stimulation trigger is recorded and when stimulation is actually provided to the E population, more about its interpretation in Sect. 7). Stimulation is implemented directly in the Euler update of our integration scheme. We aim at reproducing tremor dynamics and fit to three dynamical features: the power spectrum density (PSD) of the data, its Hilbert envelope probability density function (PDF), and its Hilbert envelope PSD. While the envelope PDF captures the range of amplitudes present in the tremor, the envelope PSD describes how quickly tremor amplitude changes. But we also aim at reproducing response to stimulation, and fit to the patient bPRC. The data dynamical features are obtained after filtering and z-scoring the data as described in Sect. 2.1. The data bPRC is obtained as described in Sect. 2.1.

**Table 2:** Best parameters for the three fitted patients

Parameter	Symbol	Best fit values		
		Patient 1	Patient 5	Patient 6
I to E weight	$w_{IE}$	9.4014	26.048	5.2064
E to I weight	$w_{EI}$	9.6306	25.3384	24.4813
E to E weight	$w_{EE}$	6.7541	1.548	2.7514
Sigmoid steepness parameter	*β*	1.1853	2.4234	4.1933
Time constant (s)	*τ*	0.0758	0.29984	0.2513
Constant input to E	$\theta_{E}$	1.4240	22.8621	2.9127
Constant input to I	$\theta_{I}$	−3.2345	−9.9279	−3.4008
Noise standard deviation	*ζ*	0.0457	0.013707	0.0263
Stimulation magnitude	*δE*	0.001684	0.00598	0.001686
Stimulation delay (ms)	$\Delta t_{\text{stim}} $	138.8366	444.1573	183.4711

**Figure 6:**
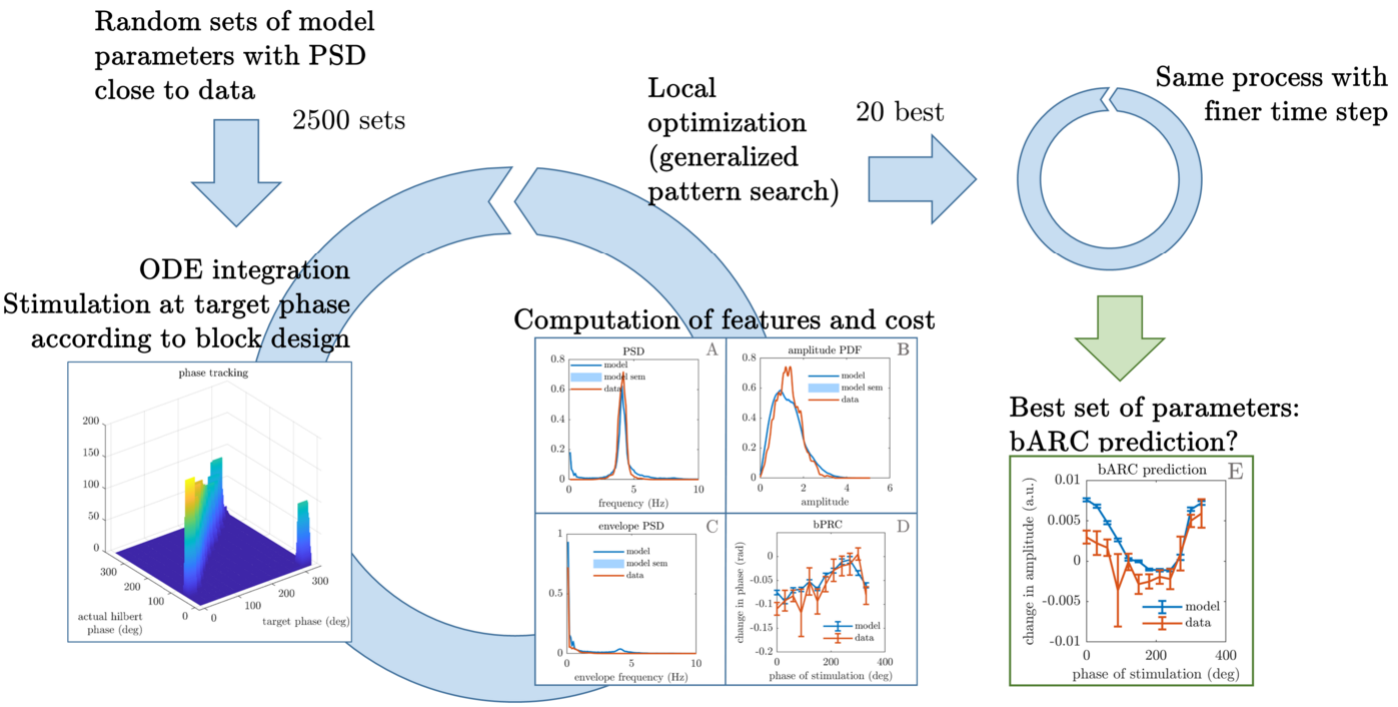
The fitting procedure involves 2500 local optimisations for each patient. The simulation of the model at each optimisation step requires one to track the zero-crossing phase in order to provide stimulation at the right phase. The phase-tracking ability of the scheme is satisfactory when compared to the actual Hilbert phase (left, detailed in Supplementary Fig. 2 in Appendix I). The optimiser minimises a cost function that includes the comparison of three tremor dynamics features (tremor PSD, tremor envelope PSD, tremor envelope PDF) plus the bPRC against the data (middle). Response curves are obtained the same way for the data and the model. Following a second optimisation of the 20 best results with a finer time step, a best set of parameters comes out of the procedure, and the model bARC can be compared against the data bARC. More details on the fitting procedure are given in Appendix F

**Supplementary Figure 2:**
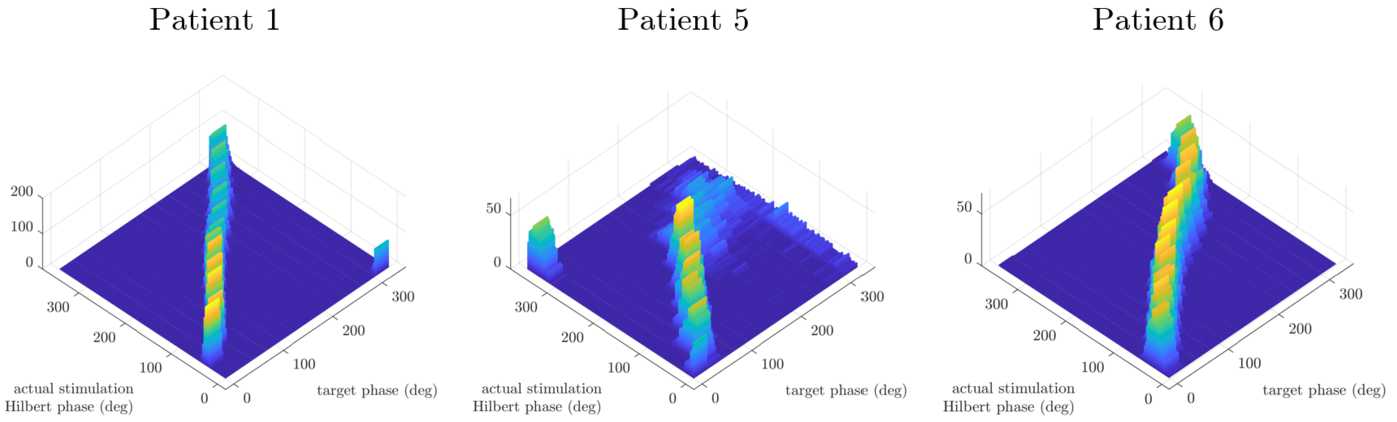
Phase tracking illustrated in the three fitted patients by histograms of the pair (target stimulation phase for the stimulation block, average of actual Hilbert phase at stimulation for the stimulation block). The actual Hilbert phase is obtained post hoc after filtering. A block average includes averaging across bursts and within the block. Averages are obtained using circular means. The effect of the stimulation delay was removed, and phases are reference to positive zero-crossings. Phase tracking is satisfactory for all patients, although tracking is less precise for later phases in patient 5

The fitting procedure is summarized in Fig. 6. Local optimisations are carried out using gradient free optimisation, specifically a direct search algorithm called the generalized pattern search algorithm (more details are given in Appendix F). In order to measure response to stimulation as in the data, each local optimisation step needs to simulate the model with phase-locked blocks of stimulation. This requires integrating the differential equations of the model while tracking the phase and providing stimulation at the right time, which is done by monitoring the zero-crossing phase alongside a Euler–Maruyama integration scheme. Appendix G details implementation of the simulator. The four features (PSD, envelope PDF, envelope PSD, bPRC) are computed on the model output at each optimisation step. The same method is used as for the data features, with three differences. The first is that for increased stability of the optimisation, the model bPRC is averaged over a much greater number of trials (600 trials), while the more robust dynamical features are obtained from nine trials only to reduce computation cost. The second is that the model output is not filtered to compute the dynamical features (only z-scored), as we want the model output to primarily generate the filtered tremor signal (a model generating mostly 1 Hz oscillations but reproducing patient tremor when filtered at 5 Hz would not be desirable). Computing the bPRC still requires filtering, as it relies on the Hilbert transform. The third difference is that the filtering window for the bPRC cannot be adjusted manually in optimisation steps, so a 4 Hz band centered on the model PSD peak is used. As for the data bPRC and bARC, the actual Hilbert phase at which stimulation occurred is used to compute response curves via the re-binning process described in Sect. 2.1, and the zero-crossing phase is only needed to enable phase-locked stimulation in the model. Phase-tracking performance is illustrated in Supplementary Fig. 2 in the Appendix.

At each step, once the four features have been computed on the model output, the optimiser returns the cost

22 $$ c = \frac{1}{4}\sum_{n = 1}^{4} \biggl( \frac{\sum_{i = 1}^{N_{n}} (y_{n,i}^{\mathrm {data}}-y_{n,i}^{\mathrm{model}} )^{2}}{\sum_{i = 1}^{N_{n}} (y_{n,i}^{\mathrm{data}}-\overline{y_{n}^{\mathrm{data}}} )^{2}} \biggr), $$

with $y_{n}, n \in\{1, 2, 3, 4\}$ being the four features considered, $N_{n}$ the length of $y_{n}$, and $\overline{y_{n}^{\mathrm{data}}}$ the mean of data feature *n*. At the end of the procedure, the fit with the highest $R^{2} = 1 - c$ for each patient is deemed the best fit. In the case of a tie (difference in mean costs lower than standard error of the mean), foci are preferred over limit cycles. The bifurcation structure of the original WC model has been studied in [36], but we simply differentiate between parameters giving rise to stable foci and limit cycles by forward simulating the model without noise, and exploring the region of phase space that is occupied by the system with noise.

### Results of the fits

**Figure 7:**
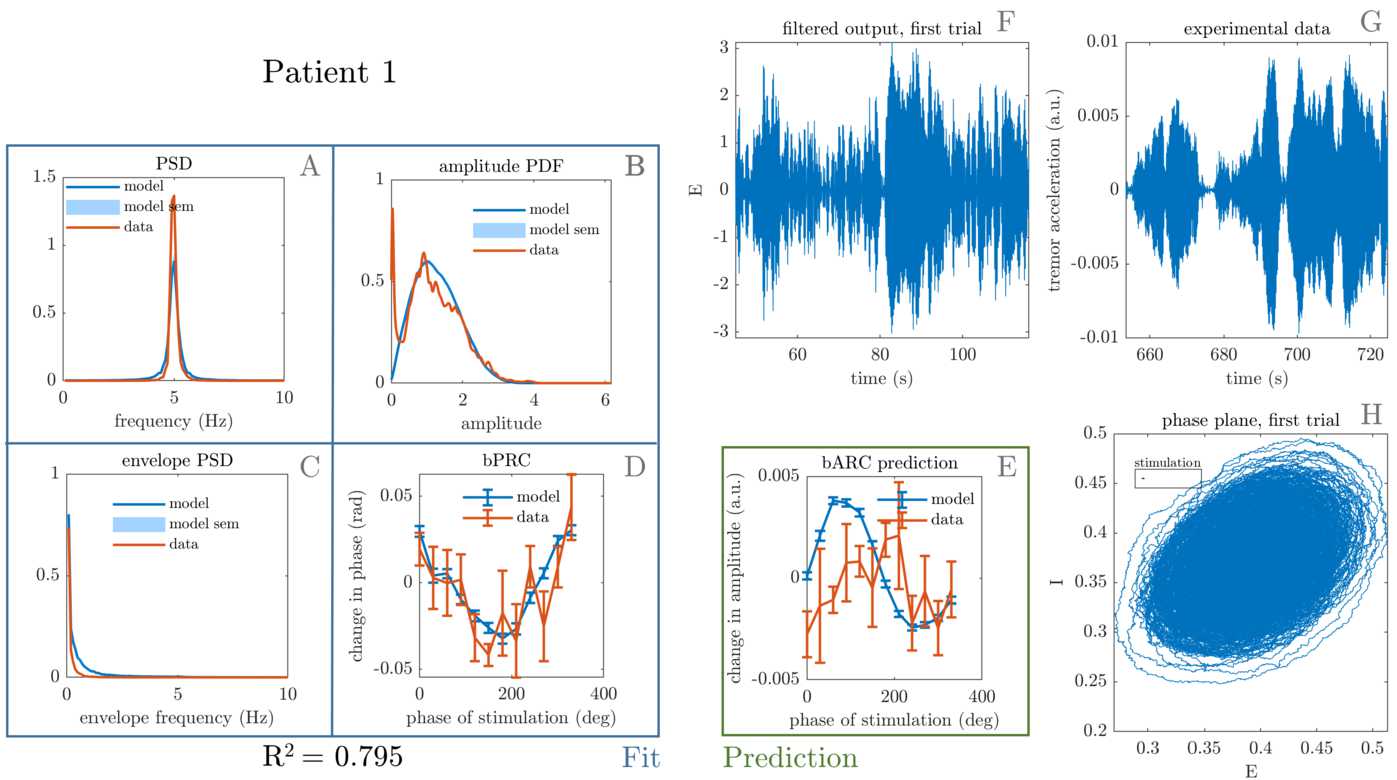
Best fit to patient 1. The four features that were included in the cost function are shown on the left, namely tremor PSD (**A**), tremor envelope PDF (**B**), tremor envelope PSD (**C**) and bPRC (**D**). The $R^{2}$ for the model fit to these features is 0.795, and the model reasonably predicts the data bARC (**E**). The model phase plane is shown in (**H**), and the model tremor time series (**F**) is shown next to the patient tremor time series (**G**). The framed black bar in (**H**) indicates the fitted stimulation magnitude to scale

**Figure 8:**
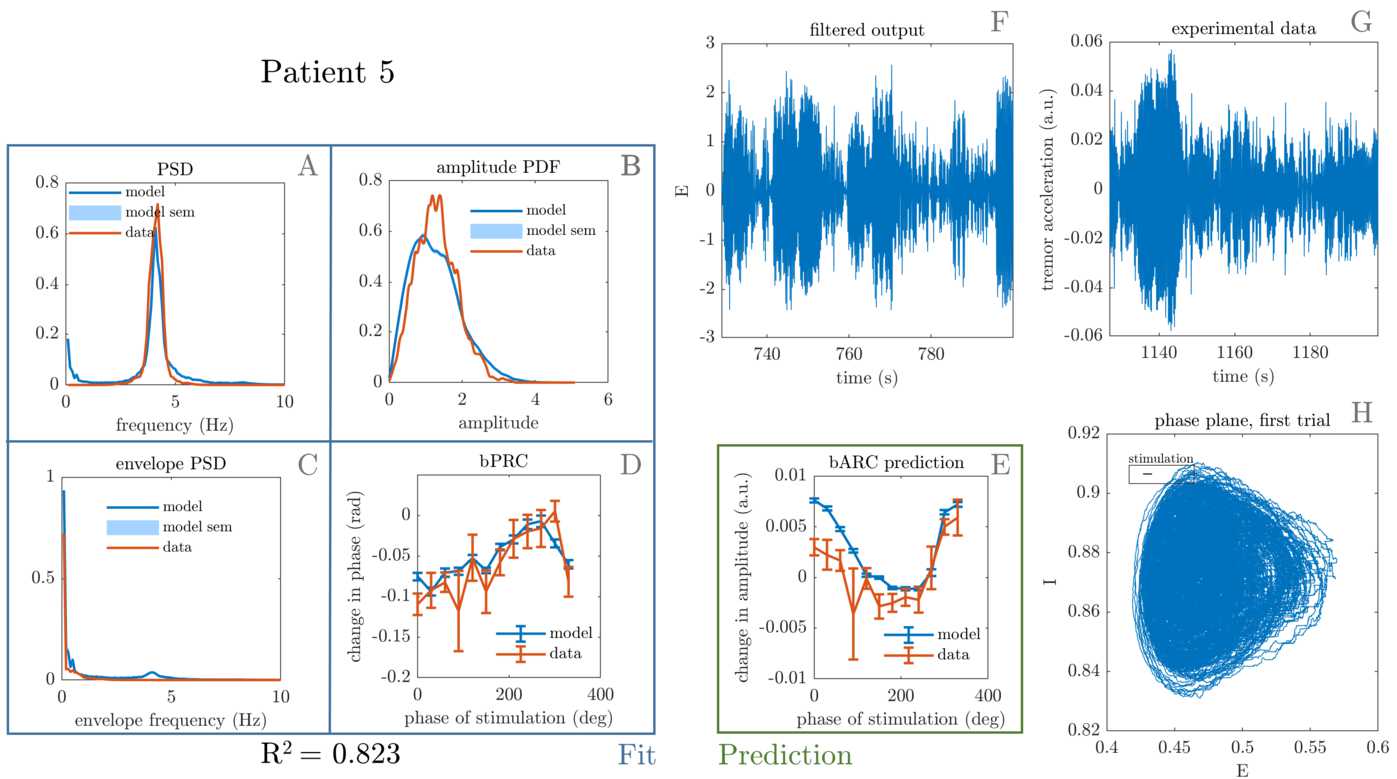
Best fit to patient 5. The four features that were included in the cost function are shown on the left, namely tremor PSD (**A**), tremor envelope PDF (**B**), tremor envelope PSD (**C**) and bPRC (**D**). The $R^{2}$ for the model fit to these features is 0.823, and the model predicts the data bARC (**E**). The model phase plane is shown in (**H**), and the model tremor time series (**F**) is shown next to the patient tremor time series (**G**). The framed black bar in (**H**) indicates the fitted stimulation magnitude to scale

**Figure 9:**
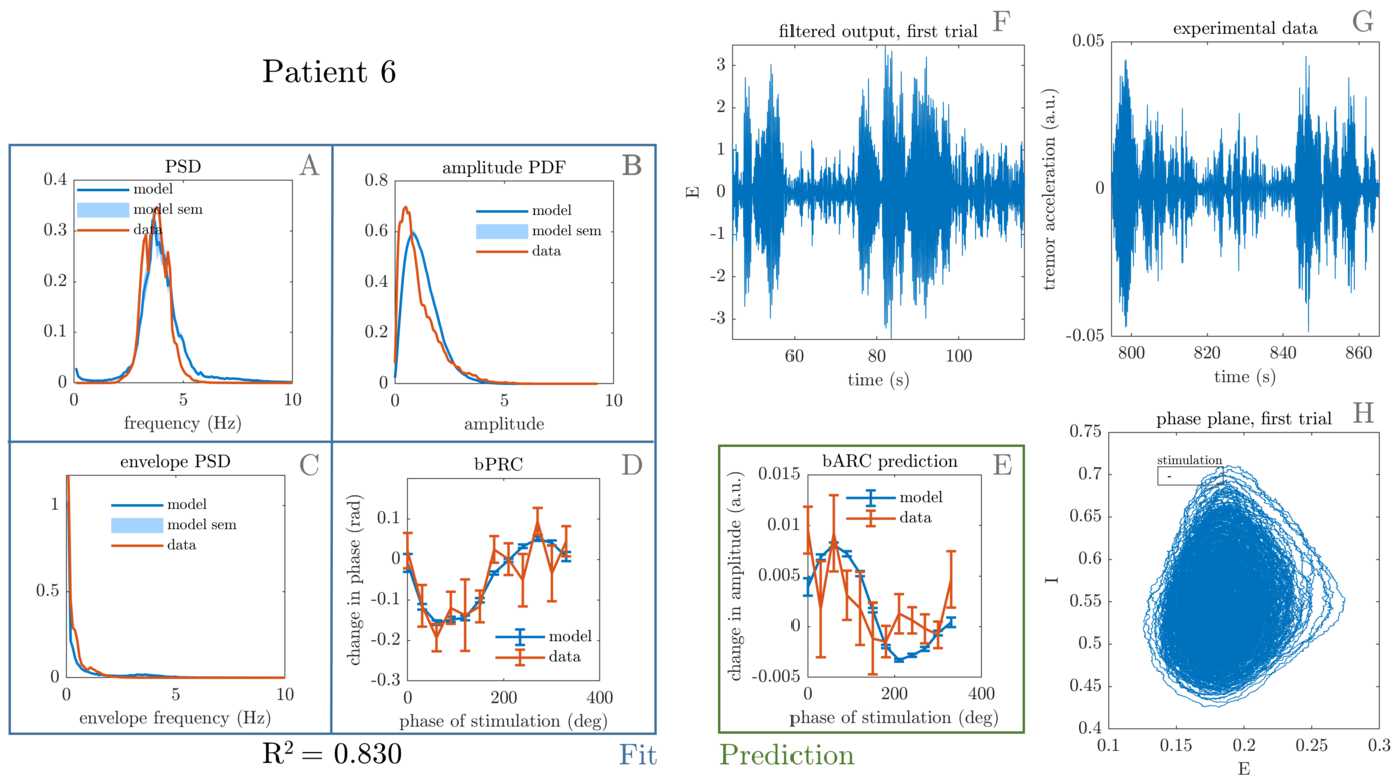
Best fit to patient 6. The four features that were included in the cost function are shown on the left, namely tremor PSD (**A**), tremor envelope PDF (**B**), tremor envelope PSD (**C**) and bPRC (**D**). The $R^{2}$ for the model fit to these features is 0.830, and the model reasonably predicts the data bARC (**E**). The model phase plane is shown in (**H**), and the model tremor time series (**F**) is shown next to the patient tremor time series (**G**). The framed black bar in (**H**) indicates the fitted stimulation magnitude to scale

Patients with both of their response curves statistically significant (see significance criterion in Sect. 2), in other words with meaningful response curves, are fitted to. For these patients, namely patient 1, 5, and 6, we find that the model successfully reproduces tremor dynamics, including tremors with sudden bursts, and can fit to patient phase response to stimulation. The best fits obtained upon completion of the optimisation procedure are shown in Figs. 7, 8, and 9. In addition to reproducing tremor dynamics and being able to fit to patient bPRCs, the model seems to be able to reasonably predict patient bARCs (obtained as in Sect. 2.1, but not fitted to), and in particular which phases are approximately the best phases to stimulate, i.e. the phases at which the maximum decrease in tremor happens. Because of averaging across 600 trials, the model bPRC and bARC error bars are small compared to the data error bars (only about 10 trials per phase bin).

#### Validating fitted stimulation magnitude

**Figure 10:**
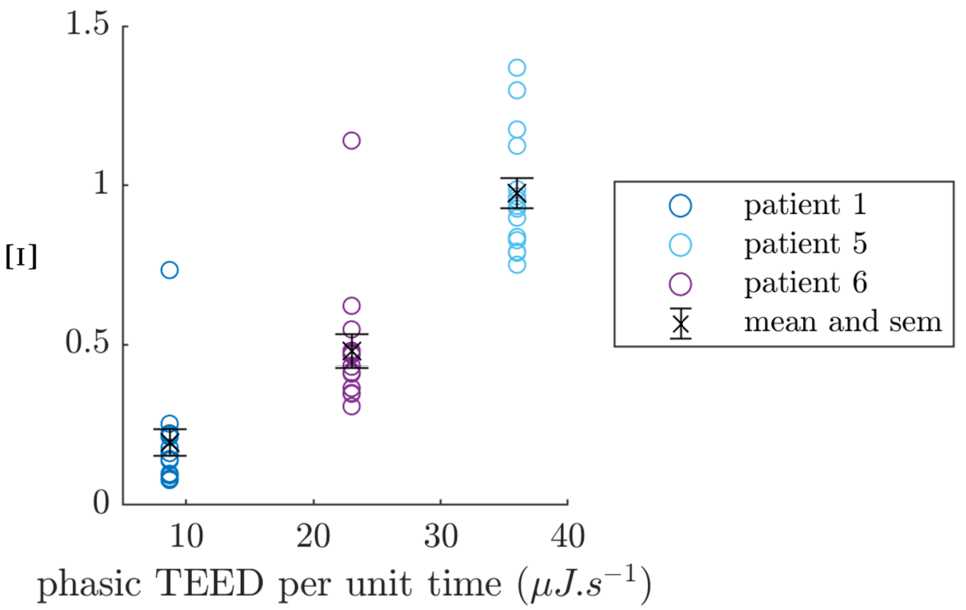
Model effective stimulation per unit time *Ξ* versus total electrical energy delivered per unit time by the device, for the three fitted patients. Showing the 15 best performing models for each patient, along with the mean and standard error of the mean error bars for each patient in black

As Cagnan et al. [11] report what the device settings are, and in particular the total electrical energy delivered (TEED) per unit time for each patient, we can validate fitted stimulation magnitudes against these values. We build an equivalent quantity *Ξ* for the model that we call “model effective stimulation per unit time”, and that should scale with the TEED per unit time. We define *Ξ* as

23 $$ \varXi= \frac{\delta E}{E_{\sigma}}\overline{f_{E}}, $$

where $E_{\sigma}$ is the standard deviation of the non z-scored first dimension of the model output, and $\overline{f_{E}}$ is the mean frequency of the first dimension of the model output. Since stimulation in the model is a direct increase in *E*, *δE* should be scaled the same way, which is the purpose of the division by $E_{\sigma}$. And since bursts are delivered once per period, the multiplication by $\overline{f_{E}}$ ensures that *Ξ* is defined per unit time (the number of pulses per burst is the same for the three patients). Figure 10 shows the model effective stimulation per unit time for the 15 best performing fits against the TEED per unit time for each patient (correlation coefficient for fit averages $r = 0.98$). Under the assumption that patient intrinsic sensitivities to stimulation are somewhat similar, we can conclude from the correlation that the fitting procedure successfully captures the scale of stimulation across patients.

#### PRC-ARC shift in WC synthetic data

**Figure 11:**
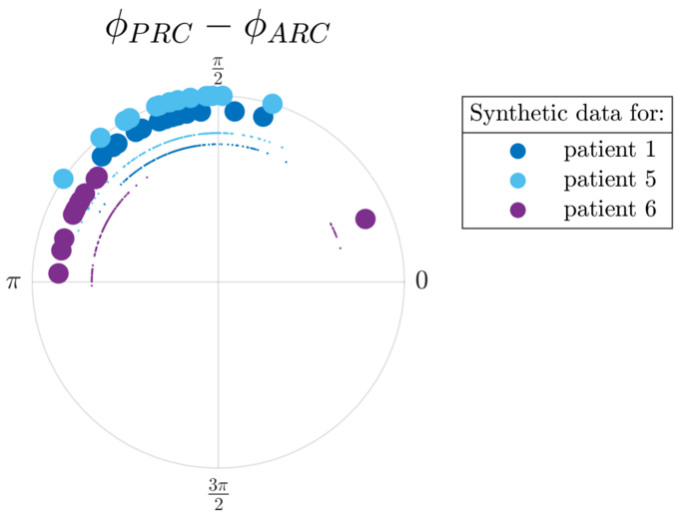
PRC-ARC shift in synthetic data (full WC model fitted to patients). For each patient, the shift for all 10 repeats of the top 15 fits is shown (smaller circles), as well as the repeat mean for each fit (larger circles). One repeat corresponds to 600 trials

The PRC-ARC shift is computed on WC synthetic data with phased-locked blocks of stimulation generated by the full model fitted to each patient. This time we can take full advantage of the model and compute bPRCs and bARCs from more trials than for patient data or model data in optimisation steps, and perform 10 repeats of 600 trials for the top 15 fits for each patient. The PRC-ARC shift is then measured as in Sect. 2.1 for each of the 10 repeats, and shown in Fig. 11. The large filled circles represent the mean of the 10 repeats for each patient fit. It appears that PRC-ARC shifts obtained for synthetic data of top patient fits mostly lie in the upper-left quadrant of the unit circle for all three patients $( [\frac{\pi}{2},\pi ] )$, similarly to patient data. One fit to patient 6 is an outlier in terms of its shift, due to high model effective stimulation (defined in the previous section). While the nonlinear model can allow for a larger shift than $\frac{\pi}{2}$, this is not the case for the linearised model, and the difference is the focus of the next section.

## PRC-ARC shift in the model

The analytical expressions for the linearised model make different predictions for patient response curves than synthetic data generated by the full model and analysed with the block method, in particular in terms of PRC-ARC shift. The present section will look at the deterministic linearisation of patient fits, and then contrast it with the full model with noise.

### Relationship between analytic response curves in the linearised fitted WC models

**Figure 12:**
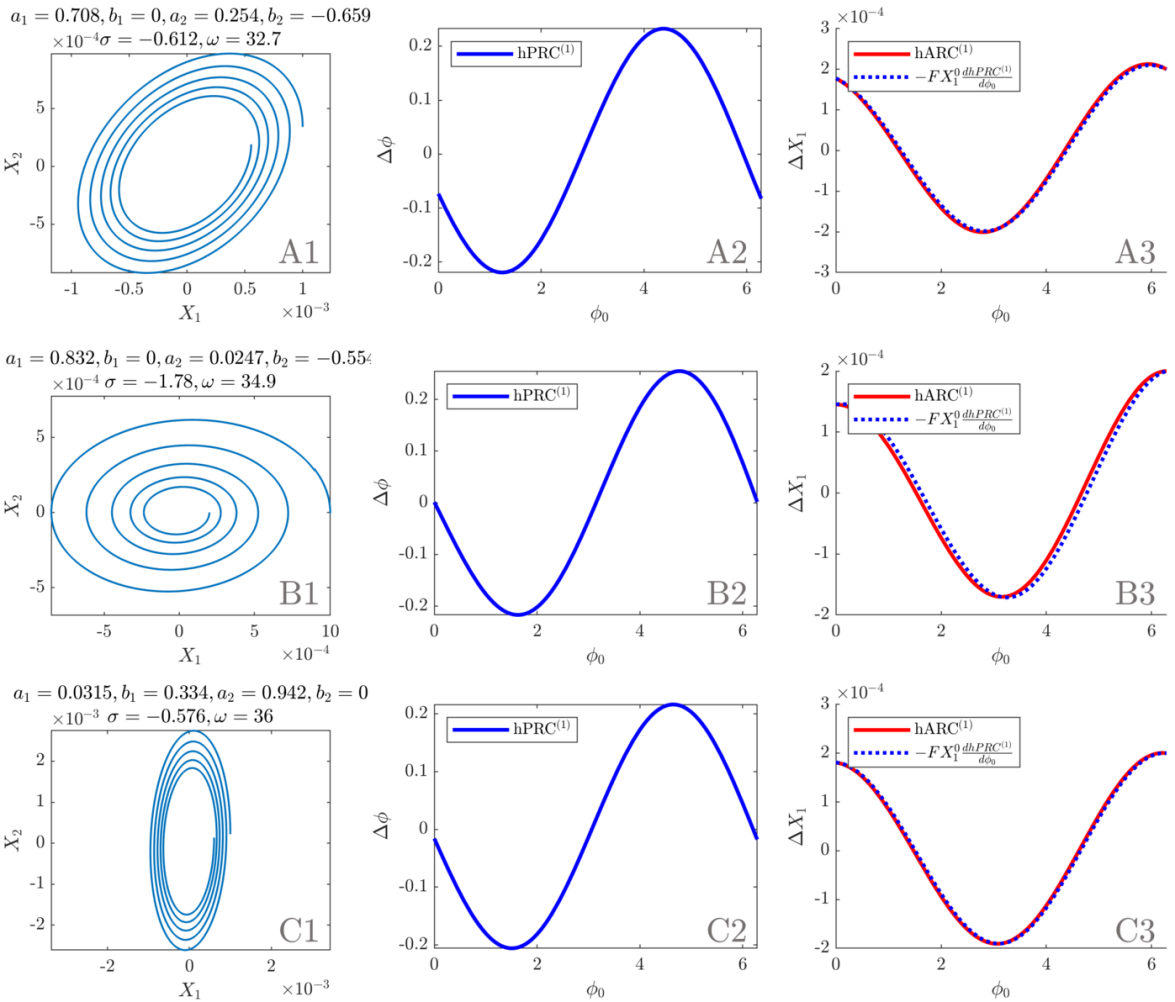
Analytical results for linearised patient fits (initial conditions as in the main text). First column: phase space. Second column: first order PRC as per Eq. (17) (scaling valid for the first cycle). Third column: first order ARC as per Eq. (19) and opposite of the derivative of the first order PRC scaled by $FX_{1}^{0}$. Panel **A**, **B**, and **C** correspond to patient 1, 5, and 6, respectively

The first order PRC and ARC expressions derived in Sect. 4 can be applied to the linearisation of the best WC models fitted to data from the three patients satisfying our significance criterion. The Jacobians at the fixed points are

$$\begin{aligned}& J_{1} = \begin{bmatrix} 11.9723 & -35.0323 \\ 34.9513 & -13.1953 \end{bmatrix} ,\qquad J_{5} = \begin{bmatrix} -0.2252 & -52.3293 \\ 23.2880 & -3.3351 \end{bmatrix} , \\& J_{6} = \begin{bmatrix} 2.8269 & -12.8784 \\ 101.6943 & -3.9789 \end{bmatrix} , \end{aligned}$$

where $J_{i}$ corresponds to patient *i*. In the fits $b_{1} = 0$ or $b_{2} = 0$, which marginally simplifies Eqs. (17) and (19). The response curves obtained are shown in Fig. 12. The same values as in Sect. 4.7 are used for $X_{1}^{0}$ and $\delta X_{1}$. Note that the stimulation delay $\Delta t_{\mathrm{stim}}$ is not shown—it affects both the PRC and the ARC and does not play a role in the PRC-ARC shift. More interestingly, we observe that $\omega\gg\vert\sigma\vert$ in the 3 fits (see Supplementary Table 2 in Appendix J), suggesting that the response curves’ relationship described by Eq. (21) should approximately hold. This is indeed the case as shown in the third column of Fig. 12, which tells us that the PRC-ARC shift should be close to $\frac{\pi}{2}$. The decay is higher for patient 5 (about 5% of the rotation versus less than 2% for the other two patients) and as expected, the approximation is slightly worse for this patient (panel B3 in Fig. 12). For small stimulation and close to the fixed point, the deterministic picture with patient parameters is that the PRC-ARC shift should be close to $\frac{\pi}{2}$. In what follows, we investigate the difference between this idealised picture and what is observed in synthetic data.

### Accounting for the difference in shift between focus model analytic expressions and WC synthetic data

Four factors could account for the difference in PRC-ARC shift between the idealised picture given by analytic response curves with patient parameters (previous section) and what is observed in WC synthetic data (Sect. 5.2). First, the stimulation may be large enough that the Taylor expansions used to derive the analytic PRC and ARC expressions are no longer approximately valid. Second, tremor in patient fits may correspond to a regime where trajectories are not so close to the fixed point, compromising the linearisation validity. Third, the introduction of noise in the model may result in effects on the PRC-ARC shift that do not average out to zero. Fourth, in synthetic data, the response to stimulation is measured by the block method, which differs from the first order approach taken in our derivations. We next show that for the three best fits considered, nonlinearity is the main driver.

Ten repeats of 600 trials of synthetic data are generated for the linearisation of the best fits to each patient. The integration scheme with live phase tracking and stimulation is the same as described in Sect. 5.1, only the stochastic differential equations are now

24 $$ \begin{bmatrix} dE \\dI \end{bmatrix} = J \begin{bmatrix} E - E^{*} \\I - I^{*} \end{bmatrix} \,dt + \zeta \begin{bmatrix} dW_{E} \\dW_{I} \end{bmatrix} , $$

where $dW_{E}$ and $dW_{I}$ are Wiener processes, *ζ* the noise standard deviation (same values as in the nonlinear case), $E^{*}$ and $I^{*}$ are the coordinates of the fixed point, and *J* is the Jacobian at the fixed point of the patient fit. The same values as in the nonlinear case are used for the stimulation magnitude and delay, with the exception of patient 5, for whom the stimulation magnitude is set to a fifth of its value in the nonlinear case, as higher values were seen to cause a breakdown of phase tracking, and result in unreliable response curves.

**Figure 13:**
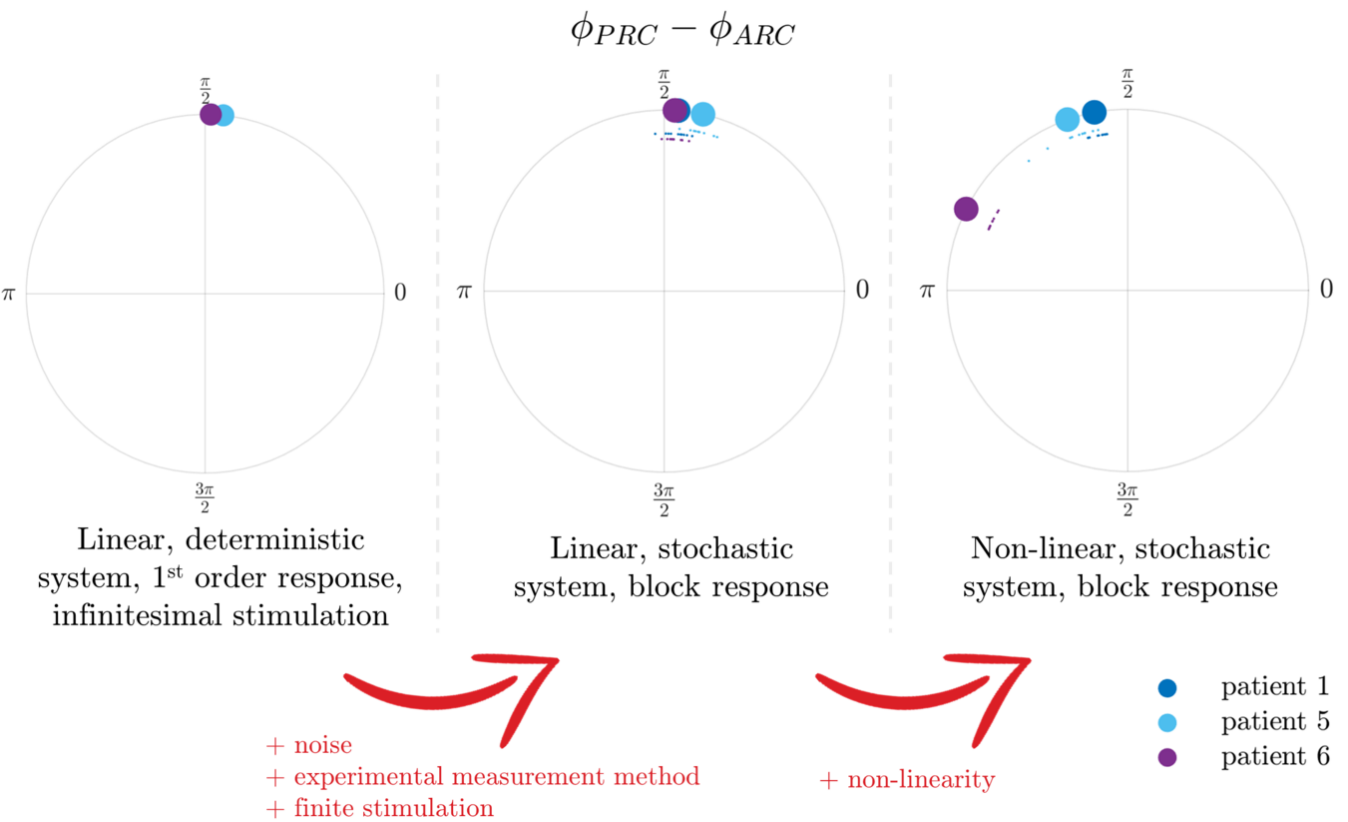
Nonlinearity accounts for most of the difference in PRC-ARC shift seen in synthetic data (middle and right), when compared to the PRC-ARC shift derived in the focus model (left). When computed from synthetic data, the PRC-ARC shift of all 10 repeats is shown (smaller circles), as well as the repeat mean (larger circles). One repeat corresponds to 600 trials, only showing the best fit for each patient

For each patient and for each of the 10 repeats, bPRCs and bARCs are obtained, and the PRC-ARC shift is then measured as in Sect. 2.1. The results are shown in Fig. 13 (middle), alongside the shifts measured from the response curves presented in Sect. 6.1 (left), and the shifts measured in the full WC model (right). It can be seen that going from the analytic response curves to the linearised model (i.e. adding noise, measuring the response to stimulation via the block method and not a first order method, and using a finite stimulation magnitude rather than a infinitesimal stimulation), does not affect the shift much (compare the left and middle panels of Fig. 13). However, a substantial increase in the shift is obtained by introducing the nonlinearity (compare the middle and right panels of Fig. 13), which brings the shift in the upper-left quadrant, where patient data lie. The PRC-ARC shift can be modulated in the nonlinear model in a way that is not available in the linearisation.

## Discussion

We showed that in a 2D linearised stable focus model, the first order PRC and ARC based on a phase definition approximately equivalent to the Hilbert phase are close to sinusoidal for small decay. Moreover, the PRC-ARC shift is close to $\frac{\pi}{2}$. Half of the patients in our dataset had significant sinusoidal bPRCs and bARCs (an effect of stimulation phase could not be found in other patients in at least one of their response curves), and the significant patients have a PRC-ARC shift in the interval $[\frac{\pi}{2},\pi ]$. A full WC model can be fitted to tremor dynamics features and to the bPRC for these patients, and as hinted at by the similarities seen in the linearised focus model and the data, the best fits—a vast majority of stable foci—can reproduce the dependence of the effects of stimulation on the phase of stimulation. The best fits also reasonably predict the bARC, and notably what is approximately the best phase to stimulate. Compared to the 2D linearised focus, the nonlinearities of the full WC model allow for a better reproduction of the phase-dependence found in patient data, in particular as far as the PRC-ARC shift is concerned. Our full model can capture the behaviour of neural populations plausibly involved in the generation of tremor, which, together with its success in reproducing phase response and predicting amplitude response in patients, makes it a strong candidate for further study of phase-locked DBS.

### Phase definition

While asymptotic phase definitions are common in theoretical studies, experimental studies tend to favour instantaneous phase definitions such as the Hilbert phase. To reproduce the data, an instantaneous phase seems more appropriate than an asymptotic phase, as there is no indication of stimulation happening on or close to an attractor. It has been shown recently in [37] how an operational definition of the phase can describe transient spiking, when an asymptotic phase does not capture the phase-dependence of transients. Moreover, stimulation is assumed to be small in our analytical expressions (Sect. 4), but not in the full model, contrary to standard asymptotic phase reduction strategies.

In this study, our phase definition is the Hilbert phase of the tremor data or approximately equivalent. It is therefore referenced to the maximum of the tremor oscillations (represented by the first coordinate of the dynamical system in our models), and does not require a limit cycle. The Hilbert phase is an angle in the analytic signal space, it does not generally grow linearly with time, and is a protophase [38]. This is not a concern from the perspective of describing patient data, as this is the observable choice we are making for both the data and the model. Commonly used with data, the Hilbert transform has also been proposed as a robust method to measure steady state PRCs in single neuron models [39].

### Linearisation

The response curves derived for the linearisation of a 2D focus in Sect. 4 can be related to previously published expressions. In particular, the infinitesimal PRC for radial isochron clocks has been derived in [40], and has been recently included in [41] under the larger umbrella of general radial isochron clocks. The radial clock case ($K(\phi)=\omega$ in [41]) perturbed along the first dimension agrees with our Eq. (17) for the case of a circular flow (see Sect. 4.7). For this simple system, the asymptotic phase response is the same as the first order Hilbert phase response.

Moreover, we demonstrated that in the linearisation of a 2D focus, the best phase to stimulate (i.e. the minimum of the ARC), corresponds to the maximum positive slope of the first order PRC (see Eq. (21)). This is valid for small decay compared to the rotation, for the phase and amplitude definitions given in Sect. 4.2 (phase approximately equivalent to the Hilbert phase, amplitude defined as the first coordinate), and for a small stimulation magnitude. In fact, the first order ARC is simply a scaled version of the opposite of the first order PRC derivative. A similar relationship has been first reported in a theoretical study in the context of an individual oscillator [42], and more recently in [15] in the context of population responses arising from the individual responses of coupled phase oscillators, whose time evolution follows Kuramoto equations, and where the level of synchrony takes the role of amplitude. The results in [15] also assume particular distributions of oscillator frequencies. It is noteworthy that we found a similar result with very few assumptions on the dynamics: our result is valid for the linearisation of any 2D focus with slow decay, i.e. any linearisation obeying Eq. (9) with slow decay. This applies in particular for the linearisation of the WC model, another popular neuroscience model very different in essence from coupled oscillator models. In the thermodynamic limit and under certain assumptions about the distribution of oscillator frequencies, the Kuramoto model can be reduced to a two-dimensional system [43, 44]. Our results are applicable to the linearisation of a fully desynchronised reduced Kuramoto model observed through $X_{1} = \rho\cos{\theta}$ where $\mathbf {r} = \rho e^{i\theta}$ is the order parameter (*ρ* is the modulus and *θ* the angle in the complex plane). Such a system therefore satisfies Eq. (21) as well (for small decay).

Our derivations do not assume proximity to a limit cycle, and this allows the study of the dependence of the response to stimulation on the amplitude of the oscillations for a given model (limit cycles do not have an amplitude variable in the case of infinitesimal perturbations). In the linearisation, the PRC is found to be inversely proportional to the amplitude of the oscillations before stimulation (see $X_{1}^{0}$ term in Eq. (17)), while the ARC does not depend on it.

Because the block method phase and amplitude response used in the rest of paper are normalised by the number of pulses and blocks are only about 25 period long, it seems legitimate to think that, although they are different objects, the first order response to a single pulse ($\mathrm{hPRC}^{(1)}$ and $\mathrm{hARC}^{(1)}$) and the block method response (bPRC and bARC) could be related, and in particular that they might have similar PRC-ARC relationships. Part of the connection hinges on our proof that the phase definition in the linearisation of the focus model matches with the Hilbert phase when the decay is small compared to the rotation (Sect. 4.2). And indeed, the PRC-ARC shift predicted by our expressions derived for the first order response to one pulse of stimulation in a linearised focus is very close to the shift obtained by the block method on linearised WC synthetic data (compare the left and middle panels of Fig. 13). Our analytical derivations provide a rationale to fit the full WC model to data and an intuition for why the model can predict patient ARC, but do not offer an exact analytic treatment of the block method. Specifically, individual pulses in a block may have different effects depending on where they are located in the block and depending on stimulation history within the block [11].

To the best of our knowledge, there is no simple way of getting analytical PRCs and ARCs based on Hilbert phase and amplitude or equivalent in the nonlinear system, making the analytical expressions for the linearisation more valuable. It is also of interest to understand what can be achieved with a simple, linear model before adding more complexity. In fact, realising that the linear model can explain already the data to some extent is a motivation to fit the nonlinear model, which is an expansive endeavour.

### Fitting procedure

Fits of the nonlinear WC model were performed using the generalized pattern search algorithm on many sets of random initial parameters. This approach was chosen for its robustness and computational efficiency in a non-smooth, non-convex landscape with four nonlinear features and 10 parameters, despite requiring the use of a supercomputer. In particular it has been deemed superior to the simplex algorithm in finding better fits. The implementation used also has the additional benefit of being able to handle failed simulations (which occasionally happen as response curves with 12 phase bins cannot be obtained for some parameter sets with noise values too high compared to the vector field). However, the fitting procedure results in many “good” local optima. What these “good” sets of parameters have in common and what they can tell us about the patients we are fitting to is not easily addressed with our current fitting strategy. Even real biological networks may have redundancies, and may exhibit the same behavior under different network configurations. Approximate Bayesian computation [45, 46] allows one to approximate the posterior distribution over parameters for intractable likelihoods, hence to answer the question what is the space of parameters consistent with the data. Whether approximate Bayesian computation methods could successfully tackle a complicated landscape and provide more meaningful insight on fitted model parameters in the setting of the present work is an interesting avenue for further research. A limitation of our fitting method is related to the integration scheme: to reduce computation cost, the Euler step used in the first optimisation process is 1 ms. The top 20 best fits are then re-optimised based on a Euler step of 0.1 ms, and results are produced with this finer time step, as dynamics can be qualitatively different (further reduction in the Euler step has not been seen to change the dynamics). While the need to track the phase at each integration step to decide if stimulation has to be applied precludes the direct use of built-in, powerful integration schemes, a more advanced custom event-based stochastic integration scheme could remove the need for a second optimisation while keeping the computation cost down. The performance of our simple phase-tracking strategy is good for patient 1 and 6 and satisfactory for patient 5 (see Supplementary Fig. 2 in the Appendix). Response curves are obtained based on the actual Hilbert phase of stimulation in a post hoc manner, which makes up for the reduced performance observed for patient 5. Still, more accurate algorithms could be explored. Our simple live phase estimation strategy is based on a linear phase evolution between zero-crossings of *E* (details in Appendix G), and it would benefit from a better frequency estimate for the current period (currently simply based on the duration of the previous period) and more robustness to noise. Even better live estimates of the Hilbert phase could be obtained thanks to autoregressive forward prediction [47], but at the expense of a higher computational cost, and of a need to adjust hyper parameters for each time series.

### Nonlinear WC model

The fitting procedure discussed above was applied to fit to data the full WC model with Gaussian white noise (Eq. (6)). The best performing fits are stable foci for all three patients, and very few limit cycles are found in the top 15 fits for all three patients. One is found for patient 1 (shares the 1st place with a stable focus—distance between mean costs only 30% of the standard error of the mean), one for patient 5, and none for patient 6. In the stable focus regime, noise brings the system away from the stable fixed point, and the interaction of the noise with the dynamics of the system makes the reproduction of patient tremor possible. In our study, noise corresponds to contributions that are not modelled by either the E/I populations or the inputs to these populations. We are considering that these contributions have no explanatory power, and model them with uncorrelated noise. While in the absence of noise, the system would converge to the stable fixed point and no tremor would be generated, Gaussian white noise cannot generate realistic tremor time series. Symptoms in the model depend just as much on the noise as on the other parameters of the model. This is shown in Appendix H where an expression is obtained for the stationary standard deviation of the linearisation of the WC model. The standard deviation is dependent on the noise, but also on the other parameters of the model via the Jacobian at the fixed point. A limitation of our approach is that comparison of the fitted weights or fitted inputs across patients may be difficult when noise levels are not comparable. Enforcing a constant level of noise in the fits or limiting noise to the minimum level required to reproduce the data may address this point. Instead of noise, tremor-like activity may be obtained by exploiting chaotic dynamics arising from coupling several WC models together [48], but this would significantly increase the complexity of the model (more on increasing complexity in the last part of this section).

Contrary to weights, stimulation delays can more easily be compared across patients, and the fitted values obtained deserve some discussion. In fitting our thalamic model to tremor acceleration, we are assuming thalamic activity and tremor are directly related as mentioned before (see Sect. 5.1). Tremor activity is, however, expected to lag thalamic activity due to conduction delays. The accelerometer used to measure tremor is also expected to introduce an electromechanical coupling delay. In the model, we allow for a stimulation delay $\Delta t_{\mathrm{stim}}$ between the stimulation trigger and the time when stimulation is actually delivered to the excitatory population. This parameter is fitted to the data, and gives the model the ability to shift its bPRC in phase. Fitted stimulation delays are hundreds of milliseconds, and conduction and accelerometer delays (tens of milliseconds) only account for a small part. The higher fitted values are required by the model to match data bPRCs. With our candidate VIM/nRT mapping in mind, the higher fitted values remain unexplained on the biology side, although as mentioned before tremor generation and ET DBS are not fully understood. It is interesting to note that the stimulation delay of the best performing model for patient 5 is longer than one period (see Table 2). This is found consistently in the top three best fits, and reducing the delay to its value modulo the average period substantially reduces the quality of the bPRC fit. Besides this short-term delay, our model does not include medium- or long-term plasticity effects, which are not expected to be strongly present in the recordings as stimulation is only delivered for periods of 5 seconds in a row. In our model, stimulation is provided to the *E* population via a direct increase in the population activity. While stimulation is provided via the sigmoid function of the excitatory population in other studies [18], we found this approach too restrictive due to sigmoid saturation, and inadequate to reproduce the full extent of the response to phase-locked DBS in some patients. As a reminder, the choice of stimulating the excitatory population rather than the inhibitory population is made for biological consistency, as the VIM is the most common stimulation target in ET DBS.

**Supplementary Figure 3:**
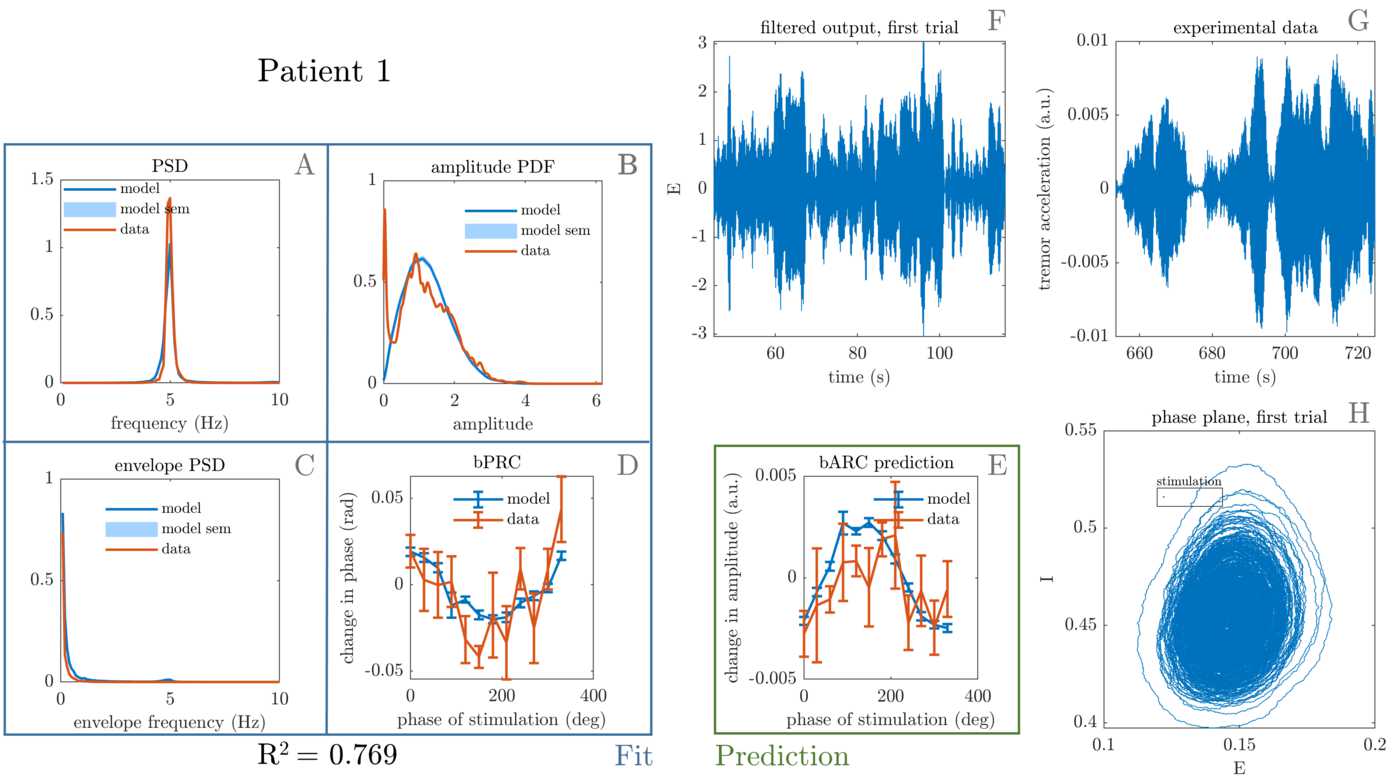
Fit to patient 1 showing the best PRC-ARC shift. The four features that were included in the cost function are shown on the left, namely tremor PSD (**A**), tremor envelope PDF (**B**), tremor envelope PSD (**C**) and bPRC (**D**). The model better predicts the data bARC (**E**) thanks to a PRC-ARC shift closer to that of the data (equivalent PRC alignment between the model and the data, but better ARC alignment). The model phase plane is shown in (**H**), and the model tremor time series (**F**) is shown next to the patient tremor time series (**G**). The framed black bar in (**H**) indicates the fitted stimulation magnitude to scale

The success of the nonlinear WC model in predicting patient ARCs when fitted to their PRCs is partially explained by its ability to modulate the PRC-ARC shift. The PRC-ARC shift in the full model can reach the range found in patients while the linearised version of the WC is limited to the close vicinity of $\frac{\pi}{2}$. The response curves of the full WC model are also better at reproducing the data and can deviate from pure sinusoids. However, there is still some room for improvement in reproducing the shift, in particular as far as patient 1 is concerned (patient shift quite a bit larger than the model). The model can allow for a larger shift as shown by a fit hand-picked in the top 15 shown in Supplementary Fig. 3 in Appendix I. While the troughs of the model bPRCs are roughly aligned with the troughs of the data bPRCs in Supplementary Fig. 3 and in our best fit in Fig. 7, it is apparent that the peaks of the model bARCs are closer to the peaks of the data bARCs in Supplementary Fig. 3 than in Fig. 7. This highlights that the PRC-ARC shift of the model is closer to that of the data in Supplementary Fig. 3 than in Fig. 7. The PRC-ARC shift could be selected as an additional feature to fit to in order to improve ARC reproduction.

In its two-population version, the suggested mapping of the excitatory and inhibitory populations (VIM and nRT) is not the only possibility. Other candidates include antidromically stimulated structures at the cerebellar level or below, such as DCN as the inhibitory population, and the inferior olive as the excitatory population. The model could also be extended by including more populations. With our current mapping in mind, the cortex and the DCN could be turned into populations of their own, which would make the model four-dimensional. As suggested in [18], the inferior olive which provides input to the DCN could also be modelled, and the spatial extent of the VIM could be accounted for by splitting it in two populations or more. Increasing the number of populations would, however, increase the number of parameters of the model, and make the optimisation process more computationally intensive, and the model more prone to over-fitting. In contrast, the incorporation of additional loops in the model architecture may help explain the inertia in stimulation effects discussed above. Nevertheless, the model seems to be able to reproduce the data in its current state, which suggests an increase in complexity is not warranted. It is remarkable that one excitatory/inhibitory loop seems to be enough to model the phase-dependent effects of ET DBS in the datasets available with statistically significant response curves. It gives some support to the hypothesis that sub-circuits of the central tremor network may behave as individual oscillators entraining each other [49].

## Conclusion

The nonlinear focus WC model with noise can reproduce the phase-dependence of the response to phase-locked DBS in ET patient data with statistically significant response curves, as well as predict tremor reduction in response to phase-locked stimulation. Phase-locked stimulation promises less stimulation, hence less side effects for the same clinical benefits, which would be highly desirable for patients. Our study positions the WC as a strong candidate to model the effects of phase-locked DBS. Its ability to describe all patients with both response curves statistically significant in at least one of our tests should be re-assessed as more data becomes available, both in terms of number of patients and recording length. Phase-dependent activity is thought to play a central role in physiological information processing [50, 51], and in our analytical derivations, the phase of the linearised model was defined in a way that does not depend on modelling oscillations by a limit cycle, and that for small decay approximately matches with a phase definition widely used in experiments, the Hilbert phase. Finally, as far as ET generation is concerned, we showed that a single excitatory/inhibitory loop is enough to reproduce both the dynamics of the tremor and the phase-dependent effects of stimulation, however, it should be nonlinear.

**Appendices**

We include here technicalities on approximating the Hilbert phase in the linearisation (Appendix A), details of the derivations leading to response curves analytical expressions in the linearised system (Appendices B to D), and the procedure used to obtain WC parameters from a given Jacobian (Appendix E). We also present details of the two-step optimisation used for fitting to patient data (Appendix F), the implementation of live-phase tracking and stimulation (Appendix G), as well as an analytical expression for the standard deviation of the tremor for the stationary linearised model (Appendix H). Supplementary figures and supplementary tables make up Appendix I and Appendix J, respectively.

## Figures and Tables

**Figure 14:**
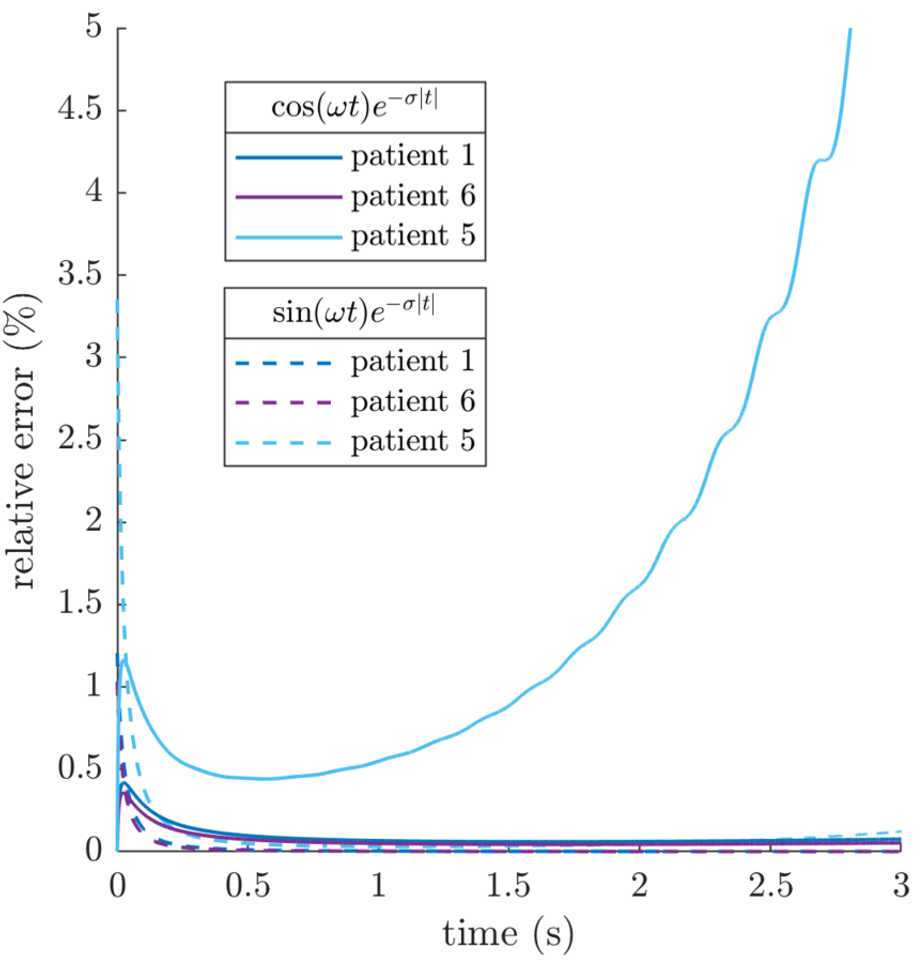
Relative error made across patients in estimating $\mathcal{H}(s(t)s_{c}(t))$ by $s(t)\mathcal{H}(s_{c}(t))$ (solid lines) and $\mathcal{H}(s(t)s_{n}(t))$ by $s(t)\mathcal{H}(s_{n}(t))$ (dashed lines). The error is calculated as the ratio of $\mathcal{I}_{s_{c}}$ (respectively, $\mathcal{I}_{s_{n}}$) over the modulus of the numerical Hilbert transform of the signal, which is the envelope of the signal. The relative error is under 5% in all cases for at least 12 periods

## Appendix 1: Hilbert transforms of sine and cosine exponential decays with error terms

The goal here is to show that $\mathcal{H}(s(t)s_{j}(t)) \approx s(t) \mathcal{H}(s_{j}(t))$ for $j=c,n$, with $s(t)=e^{\sigma\vert t \vert}$, $s_{c}(t)=\cos{\omega t}$, and $s_{n}(t) = \sin{\omega t}$. The Bedrosian identity [52] states that the Hilbert transform of the product of a low-pass and a high-pass signal with non-overlapping spectra is the product of the low-pass signal and the Hilbert transform of the high-pass signal. The spectrum support of *s* is $\mathbb{R}$ and therefore overlaps with the spectra of $s_{c}$ and $s_{n}$, but for low decay compared to the rotation, the spectrum of *s* is very small where it overlaps with the spectra of $s_{c}$ or $s_{n}$. Because of the overlaps, the equality given by the Bedrosian identity is not exact and turns into an approximation, and inspired by the proof in [52], we can calculate error terms. Let *S* and $S_{c}$ be the Fourier transforms of *s* and $s_{c}$, respectively:

25 $$\begin{aligned}& s(t)s_{c}(t) = \frac{1}{(2\pi)^{2}} \int_{-\infty}^{\infty} \int_{- \infty}^{\infty}S(u)S_{c}(v)e^{i(u+v)t}\,du\,dv, \end{aligned}$$

26 $$\begin{aligned}& \mathcal{H}\bigl(s(t)s_{c}(t)\bigr) = \frac{1}{(2\pi)^{2}} \int_{-\infty}^{ \infty} \int_{-\infty}^{\infty}S(u)S_{c}(v)i \operatorname {sgn}(u+v)e^{i(u+v)t}\,du\,dv. \end{aligned}$$

The Fourier transform of $s_{c}$ is given by $S_{c}(v) = \pi [\delta(v-\omega)+\delta(v+\omega) ]$, so

27 $$ \mathcal{H}\bigl(s(t)s_{c}(t)\bigr) = \frac{1}{(2\pi)^{2}} \int_{-\infty}^{ \infty}S(u)e^{iut}\varGamma(u)\,du, $$

where $\varGamma(u) = \frac{\pi}{i} [\operatorname {sgn}(u+\omega)e^{i\omega t} + \operatorname {sgn}(u-\omega)e^{-i\omega t} ]$. This can be simplified as

$$ \varGamma(u) = 2\pi\sin{\omega t} + \textstyle\begin{cases} 0, & \vert u \vert < \omega, \\ - \frac{2\pi}{i}e^{i\omega t}, & u < -\omega, \\ \frac{2\pi}{i}e^{-i\omega t}, & u > \omega. \end{cases} $$

The Fourier transform $S(u)=\frac{2\sigma}{\sigma^{2}+u^{2}}$ is even, therefore

28 $$\begin{aligned}& \mathcal{H}\bigl(s(t)s_{c}(t)\bigr) = \frac{\sin(\omega t)}{(2\pi)^{2}} \int_{-\infty}^{\infty}S(u)e^{iut}\,du + \frac{1}{2\pi i} \int_{ \omega}^{\infty}S(u) \bigl(e^{i(u-\omega)t}-e^{-i(u-\omega)t} \bigr)\,du, \end{aligned}$$

29 $$\begin{aligned}& \mathcal{H}\bigl(s(t)s_{c}(t)\bigr) = s(t)\mathcal{H} \bigl(s_{c}(t)\bigr) + \mathcal{I}_{s_{c}}, \end{aligned}$$

with

$$\begin{aligned}& \mathcal{H}\bigl(s_{c}(t)\bigr) = \sin{\omega t}, \\& \mathcal{I}_{s_{c}} = \frac{2}{\pi} \int_{\omega}^{\infty} \frac{\sigma}{\sigma^{2}+u^{2}}\sin{(u- \omega)t}\,du. \end{aligned}$$

A similar derivation provides

30 $$ \mathcal{H}\bigl(s(t)s_{n}(t)\bigr) = s(t)\mathcal{H} \bigl(s_{n}(t)\bigr) + \mathcal{I}_{s_{n}} $$

with

$$\begin{aligned}& \mathcal{H}\bigl(s_{n}(t)\bigr) = -\cos{\omega t}, \\& \mathcal{I}_{s_{n}} = \frac{2}{\pi} \int_{\omega}^{\infty} \frac{\sigma}{\sigma^{2}+u^{2}}\cos{(u- \omega)t}\,du. \end{aligned}$$

Numerical integration demonstrates that for $\omega\gg\vert\sigma\vert$, and in particular in the case of the patients we are interested in, $\mathcal{I}_{s_{c}}$ and $\mathcal{I}_{s_{n}}$ are under 5% of the signal scale for about 12 periods (see Fig. 14). This is more than enough for our purposes as only one period is needed to derive response curves. It is therefore reasonable to ignore $\mathcal{I}_{s_{c}}$ and $\mathcal{I}_{s_{n}}$.

## Appendix 2: Reference trajectory without stimulation

Let us find the coefficients $K_{\mathrm{ref}}$ and $K'_{\mathrm{ref}}$ of the trajectory starting at $t=0$ at a maximum of the first coordinate $X_{1}=X_{1}^{0}>0$. With the choice $\phi= \omega t$, this will ensure we are referencing the phase to the maximum of $X_{1}$. It should be noted at this point that we are not using the Jacobian in what follows as we are interested in the dependence of the response on the rotation *ω* and the decay *σ*. From the initial condition at $t=0$,

31 $$ K_{\mathrm{ref}} a_{1} + K'_{\mathrm{ref}} b_{1} = X_{1}^{0}. $$

Additionally, $X_{1}^{0}$ being a maximum requires that $\frac{d X_{1}}{dt}=0$ at $t=0$, therefore

32 $$\begin{aligned} \frac{d X_{1}}{dt} =& e^{\sigma t} \bigl[ -\omega\bigl(K_{\mathrm {ref}}a_{1}+K'_{\mathrm{ref}}b_{1} \bigr) \sin{\omega t} + \omega\bigl(-K_{\mathrm{ref}}b_{1}+K'_{\mathrm {ref}}a_{1} \bigr)\cos{\omega t} \\ &{}+\sigma \bigl\{ \bigl(K_{\mathrm{ref}}a_{1}+K'_{\mathrm {ref}}b_{1} \bigr)\cos{\omega t}+\bigl(-K_{\mathrm{ref}}b_{1}+K'_{\mathrm{ref}}a_{1} \bigr) \sin{\omega t} \bigr\} \bigr]. \end{aligned}$$

Using the condition at $t=0$,

33 $$\begin{aligned}& K_{\mathrm{ref}}(\sigma a_{1} - \omega b_{1})+K'_{\mathrm{ref}}( \sigma b_{1} + \omega a_{1}) = 0 , \end{aligned}$$

34 $$\begin{aligned}& \text{(31)} + \text{(33)}\quad \implies\quad \begin{aligned} &K_{\mathrm{ref}} = \frac{\sigma b_{1} + \omega a_{1}}{\omega( a_{1}^{2} + b_{1}^{2})}X_{1}^{0}, \\ &K'_{\mathrm{ref}} = \frac{-\sigma a_{1} + \omega b_{1}}{\omega( a_{1}^{2} + b_{1}^{2})}X_{1}^{0}. \end{aligned} \end{aligned}$$

We are excluding the case where the denominator in (34) is equal to zero, which corresponds to both $a_{1}$ and $b_{1}$ being zero, which would imply $X_{1}(t) = 0$. Also note that by picking a positive $X_{1}^{0}$, we are ensuring that the null derivative corresponds to a maximum of $X_{1}$ rather than a minimum.

## Appendix 3: Trajectory with stimulation

Let us determine what the coefficients $K_{\mathrm{stim}}$ and $K'_{\mathrm{stim}}$ are for the stimulated trajectory (still constrained by the dynamics of Eq. (9)). We have

35 $$ \mathbf {X^{1^{+}}}= \bigl\{ K_{\mathrm{stim}} (\mathbf {a}\cos{\phi _{0}}-\mathbf {b} \sin{\phi_{0}} ) +K'_{\mathrm{stim}} (\mathbf {a}\sin{\phi_{0}}+ \mathbf {b}\cos{\phi_{0}} ) \bigr\} e^{\sigma \frac{\phi_{0}}{\omega}}. $$

Solving for $K_{\mathrm{stim}}$ gives

36 $$ K_{\mathrm{stim}}= \frac{X_{2}^{1^{-}}(a_{1}\sin{\phi_{0}}+b_{1}\cos{\phi _{0}})-(X_{1}^{1^{-}}+\delta X_{1})(a_{Y} \sin{\phi_{0}} + b_{2} \cos {\phi_{0}})}{a_{2} b_{1} - a_{1} b_{2}} e^{-\sigma\frac{\phi _{0}}{\omega}}. $$

Plugging in $X_{1}^{1^{-}}$, $X_{2}^{1^{-}}$, and the expressions for $K_{\mathrm{ref}}$ and $K'_{\mathrm{ref}}$ yields

37 $$ K_{\mathrm{stim}}= \frac{\omega a_{1} + \sigma b_{1}}{\omega( a_{1}^{2} + b_{1}^{2} )}X_{1}^{0} - \frac{a_{2}\sin{\phi_{0}}+b_{2}\cos{\phi_{0}}}{a_{2} b_{1} - a_{1} b_{2}} \delta X_{1} e^{-\sigma \frac{\phi_{0}}{\omega}}. $$

Similarly for $K'_{\mathrm{stim}}$, using the previous result,

38 $$\begin{aligned}& K'_{\mathrm{stim}}= \frac{X_{2}^{1-} e^{-\sigma\frac{\phi_{0}}{\omega}} - K_{\mathrm {stim}}(a_{1}\cos{\phi_{0}}-b_{1}\sin{\phi_{0}})}{a_{1}\sin{\phi _{0}}+b_{1}\cos{\phi0}}, \end{aligned}$$

39 $$\begin{aligned}& K'_{\mathrm{stim}}= \frac{-\sigma a_{1} + \omega b_{1}}{\omega( a_{1}^{2} + b_{1}^{2} )}X_{1}^{0} + \frac{a_{2}\cos{\phi_{0}}-b_{2}\sin{\phi_{0}}}{a_{2} b_{1} - a_{1} b_{2}} \delta X_{1} e^{-\sigma \frac{\phi_{0}}{\omega}}. \end{aligned}$$

## Appendix 4: Phase at the next maximum of $X_{1}$ on the stimulated trajectory

We are looking for $\phi_{\mathrm{max}}$ such that $\frac{d X_{1}^{\mathrm{stim}}}{dt} = 0 $ at $\omega t = \phi_{\mathrm {max}}$. This give us

40 $$\begin{aligned}& e^{\sigma\frac{\phi_{\mathrm{max}}}{\omega}} \bigl[ -\omega \bigl(K_{\mathrm{stim}}a_{1}+K'_{\mathrm{stim}}b_{1} \bigr) \sin{\phi_{\mathrm{max}}} + \omega\bigl(-K_{\mathrm {stim}}b_{1}+K'_{\mathrm{stim}}a_{1} \bigr)\cos{\phi_{\mathrm{max}}} \\& \quad {}+\sigma \bigl\{ \bigl(K_{\mathrm{stim}}a_{1}+K'_{\mathrm {stim}}b_{1} \bigr)\cos{\phi_{\mathrm{max}}}+\bigl(-K_{\mathrm {stim}}b_{1}+K'_{\mathrm{stim}}a_{1} \bigr) \sin{\phi_{\mathrm{max}}} \bigr\} \bigr]=0, \end{aligned}$$

41 $$\begin{aligned}& \tan{\phi_{\mathrm{max}}}= \frac{K_{\mathrm{stim}}(\sigma a_{1} - \omega b_{1}) + K'_{\mathrm {stim}}(\sigma b_{1} + \omega a_{1})}{K_{\mathrm{stim}}(\sigma b_{1} + \omega a_{1}) + K'_{\mathrm{stim}}(-\sigma a_{1} + \omega b_{1})}. \end{aligned}$$

The phase $\phi_{\mathrm{max}}$ is returned by the arctan function in $(-\frac{\pi}{2},\frac{\pi}{2} )$, and corresponds to the previous peak on the stimulated trajectory extended backwards. The next peak has the same $\mathrm{phase} \pmod{2\pi}$ as the expression in square brackets in Eq. (40) is 2*π*-periodic.

## Appendix 5: Finding WC parameters corresponding to a given Jacobian

The Jacobian of (6) evaluated at $(E^{*},I^{*})$ can be simplified by making use of $f'(x)=\beta f(x)(1-f(x))$. We also have

42 $$\begin{aligned}& f(\varTheta_{1}) = E^{*} , \end{aligned}$$

43 $$\begin{aligned}& f(\varTheta_{2}) = I^{*} , \end{aligned}$$

with

$$\begin{aligned}& \varTheta_{1} = w_{EE}E^{*} - w_{IE}I^{*} + \theta_{E}, \\& \varTheta_{2} = w_{EI}E^{*} + \theta_{I}. \end{aligned}$$

The Jacobian of (6) evaluated at $(E^{*},I^{*})$ is therefore given by

44 $$\begin{aligned} J_{\mathrm{WC}} =& \frac{1}{\tau} \begin{bmatrix} w_{EE}f'(\varTheta_{1}) - 1 & -w_{IE}f'(\varTheta_{1}) \\w_{EI}f'(\varTheta_{2}) & -1 \end{bmatrix} \\ =& {\frac{1}{\tau}} \begin{bmatrix} w_{EE}\beta E^{*}(1-E^{*}) - 1 & -w_{IE}\beta I^{*}(1-I^{*}) \\ w_{EI}\beta E^{*}(1-E^{*}) & -1 \end{bmatrix}. \end{aligned}$$

We are interested in finding WC parameters so that the linearisation of the WC model at the fixed point will be characterised by a given Jacobian matrix

45 $$ J = \begin{bmatrix} J_{11} & J_{12} \\J_{21} & J_{22} \end{bmatrix} . $$

If we pick values for *β*, $E^{*}$ and $I^{*}$, the remaining parameters can be obtained by equating (44) and (45), and by re-arranging Eqs. (42) and (43). The parameters in Supplementary Table 1 were obtained using this method, which yields

$$\begin{aligned}& \tau = - \frac{1}{J_{22}}, \\& w_{EE} = \frac{\tau J_{11} + 1}{\beta E^{*} (1-E^{*}),} \\& w_{IE} = - \frac{\tau J_{12}}{\beta E^{*} (1-E^{*})}, \\& w_{EI} = \frac{\tau J_{21}}{\beta I^{*} (1-I^{*})}, \\& \theta_{E} = 1 - \frac{1}{\beta}\ln \biggl( \frac{1}{E^{*}}-1 \biggr) - w_{EE}E^{*} + w_{IE}I^{*}, \\& \theta_{I} = 1 - \frac{1}{\beta}\ln \biggl( \frac{1}{I^{*}}-1 \biggr) - w_{EI}E^{*}. \end{aligned}$$

## Appendix 6: Two-step optimisation

The optimisation procedure is as follows. For each patient, random sets of parameters are picked from uniform distributions (bounds in Supplementary Table 3). To improve the efficiency of the optimisation, we accept parameters only if the PSD peak of the corresponding model (without stimulation) is within 1 Hz and 25% in magnitude of the data PSD peak. Once 2500 parameter sets have been accepted, we put them through local optimisations. Local optimisations are carried out using a direct search algorithm called the generalized pattern search algorithm [53, 54]. The pattern is a set of fixed vectors in parameter space. At each step, points to be polled (the mesh) are generated by adding a scaled version of the pattern to the current best point. If a point with a lower value of the objective function is found, this point becomes the new best point, and a scaled up version of the pattern is used next. If not, a scaled down version of the pattern is used next. Parameters supplied to pattern search are put on a similar scale to improve search robustness, and hard limits are given to the optimiser (see Supplementary Table 3 in Appendix J). Optimisations are performed in parallel on a supercomputer. A time step of 1 ms is used for the fits (a period is about 200 ms). At the end of this process, the 20 best performing sets of parameters were put through more local optimisations with a finer time step of 0.1 ms and stop criteria leaving room for more steps. The finer time step is also used to produce the results shown in Sect. 5.2).

The implementation of the generalized pattern search algorithm used is Matlab’s patternsearch optimiser with the poll method “positive basis 2N” and the following stop criteria:

- main optimisation (time step of 1 ms): mesh size of 10^−4^, function call budget of 800,
- second optimisation (time step of 0.1 ms): mesh size of 10^−5^, function call budget of 1000.

## Appendix 7: Live phase tracking and stimulation

One simulation consists of 600 trials with 12 blocks of phase-locked stimulation each. As in the experimental paradigm, blocks last 5 s, and inter-block intervals are 1 s. Inter-trial intervals are 5 s, and the first trial starts after about 200 periods. During this initial time, the mean of *E* and the standard deviation of *E*, $\sigma_{\mathrm{sim}}$, are obtained from about 20 periods after a ramp-up of about 40 periods. Phase-tracking subsequently starts: *E* is centered and a threshold $T = 0.2\sigma_{\mathrm{sim}}$ is used to track positive zero-crossings. We define a positive zero-crossing as happening when

46 $$ \textstyle\begin{cases} E(n) < -T, \\ E(p) > T, \\ p>n, \\ \forall i \in \{ n+1,\ldots,p-1 \} ,\quad E(i) \in[-T,T]. \end{cases} $$

These conditions are constantly monitored, and if found true, a positive zero-crossing is declared to have happened at time step $\chi=\frac{n+p}{2}$. The threshold *T* was found critical to handle the noise included in the model, as it prevents situations where a negative zero-crossing very closely follows a positive zero-crossing (or vice versa) from interfering. We evolve the zero-crossing phase according to a frequency based on the previous period, and if $\chi_{k}$ is the last positive zero-crossing to have occurred, the current value of the zero-crossing phase is given by

47 $$ \varphi= \frac{2\pi}{t_{\chi_{k}}-t_{\chi_{k-1}}}(t-t_{\chi_{k}}). $$

If the value of 2*π* is reached, the phase value is set to 0 until the next positive zero-crossing is detected. Stimulation is provided after *φ* reaches the target phase for the block, and the stimulation trigger is recorded $\Delta t_{\mathrm{stim}}$ before stimulation occurs. If the zero-crossing phase has not reached the target stimulation phase yet when the next positive zero-crossing is detected, stimulation is provided right then. As in [11], a pulse of stimulation consists of six quick bursts at 130 Hz.

## Appendix 8: Stationary standard deviation of the first coordinate in the linearised model

In the absence of stimulation, the stationary covariance matrix $P^{\infty}$ of the linearised model (Eq. (24)) must satisfy (see [55])

48 $$ JP^{\infty}+ P^{\infty}J^{\mathrm{T}} + \begin{bmatrix} \zeta^{2} & 0 \\ 0 & \zeta^{2} \end{bmatrix} = 0. $$

Solving for $P^{\infty}$ and following the notation in Eq. (45), we can write the stationary standard deviation of the first coordinate in the linearised model as

49 $$ \sqrt{P^{\infty}_{11}} = \zeta\sqrt{ \frac{J_{12}^{2} + J_{22}^{2} + J_{11}J_{22} - J_{12}J_{21}}{2(J_{11}+J_{22})(J_{12}J_{21}-J_{11}J_{22})}}. $$

## Appendix 10: Supplementary tables

**Supplementary Table 1:** WC parameters corresponding to the Jacobians presented in Sect. 4.7. The steepness parameter *β* was set to 4, $E^{*}$ and $I^{*}$ to 0.5, and parameters were determined according the method presented in Appendix E

Parameter	Symbol	$J_{\text{circ}}^{\text{slow}}$	$J_{\text{circ}}^{\text{fast}}$	$J_{\text{ellip}}$
I to E weight	$w_{IE}$	200	5	1
E to I weight	$w_{EI}$	200	5	2
E to E weight	$w_{EE}$	0	0	2
Sigmoid steepness parameter	*β*	4	4	4
Time constant (s)	*τ*	200	5	1
Constant input to E	$\theta_{E}$	101	3.5	0.5
Constant input to I	$\theta_{I}$	−99	−1.5	0

**Supplementary Table 2:** $\vert\sigma\vert/\omega$ ratios in the linearisation of patient fits

	Patient 1	Patient 5	Patient 6
$\frac{\vert\sigma \vert}{\omega}$	1.9%	5.1%	1.6%

**Supplementary Table 3:** Lower and upper bounds of parameters uniform distributions used to generate initial parameters for fitting, and hard limits enforced by pattern search during the optimisation process

Parameter	Symbol	Initial parameter distribution		Hard limits enforced by optimizer	
		Lower bound	Upper bound	Lower bound	Upper bound
I to E weight	$w_{IE}$	0	10	0	30
E to I weight	$w_{EI}$	0	10	0	30
E to E weight	$w_{EE}$	0	10	0	30
Sigmoid steepness parameter	*β*	0	10	0	30
Time constant (s)	*τ*	0	0.3	0	0.5
Constant input to E	$\theta_{E}$	−2	10	−30	30
Constant input to I	$\theta_{I}$	−10	2	−30	30
Noise standard deviation	*ζ*	0	0.1	0	0.3
Stimulation magnitude	*δE*	0	0.02	0	0.1
Stimulation delay (ms)	$\Delta t_{\text{stim}} $	0	250	0	500

## Abbreviations

ARC: amplitude response curve
DBS: deep brain stimulation
DCN: deep cerebellar nuclei
ET: essential tremor
FDR: false discovery rate
nRT: reticular nucleus
PD: Parkinson’s disease
PDF: probability density function
PRC: phase response curve
PSD: power spectrum density
TEED: total electrical energy delivered
VIM: ventral intermediate nucleus
WC: Wilson–Cowan

## References

[1] Louis ED, Ferreira JJ (2010). How common is the most common adult movement disorder? Update on the worldwide prevalence of essential tremor. Mov Disord.

[2] McIntyre CC, Anderson RW (2016). Deep brain stimulation mechanisms: the control of network activity via neurochemistry modulation. J Neurochem.

[3] Kumar R, Lozano AM, Sime E, Lang AE (2003). Long-term follow-up of thalamic deep brain stimulation for essential and Parkinsonian tremor. Neurology.

[4] Børretzen MN, Bjerknes S, Sæhle T, Skjelland M, Skogseid IM, Toft M, Dietrichs E (2014). Long-term follow-up of thalamic deep brain stimulation for essential tremor—patient satisfaction and mortality. BMC Neurol.

[5] Little S, Pogosyan A, Neal S, Zavala B, Zrinzo L, Hariz M, Foltynie T, Limousin P, Ashkan K, FitzGerald J, Green AL, Aziz TZ, Brown P (2013). Adaptive deep brain stimulation in advanced Parkinson disease. Ann Neurol.

[6] Little S, Tripoliti E, Beudel M, Pogosyan A, Cagnan H, Herz D, Bestmann S, Aziz T, Cheeran B, Zrinzo L, Hariz M, Hyam J, Limousin P, Foltynie T, Brown P (2016). Adaptive deep brain stimulation for Parkinson’s disease demonstrates reduced speech side effects compared to conventional stimulation in the acute setting. J Neurol Neurosurg Psychiatry.

[7] Rosa M, Arlotti M, Ardolino G, Cogiamanian F, Marceglia S, Di Fonzo A, Cortese F, Rampini PM, Priori A (2015). Adaptive deep brain stimulation in a freely moving Parkinsonian patient. Mov Disord.

[8] Holt AB, Wilson D, Shinn M, Moehlis J, Netoff TI (2016). Phasic burst stimulation: a closed-loop approach to tuning deep brain stimulation parameters for Parkinson’s disease. PLoS Comput Biol.

[9] Brittain JS, Probert-Smith P, Aziz TZ, Brown P (2013). Tremor suppression by rhythmic transcranial current stimulation. Curr Biol.

[10] Holt AB, Kormann E, Gulberti A, Pötter-Nerger M, McNamara CG, Cagnan H, Baaske MK, Little S, Köppen JA, Buhmann C (2019). Phase-dependent suppression of beta oscillations in Parkinson’s disease patients. J Neurosci.

[11] Cagnan H, Pedrosa D, Little S, Pogosyan A, Cheeran B, Aziz T, Green A, Fitzgerald J, Foltynie T, Limousin P, Zrinzo L, Hariz M, Friston KJ, Denison T, Brown P (2017). Stimulating at the right time: phase-specific deep brain stimulation. Brain.

[12] Zirh TA, Lenz FA, Reich SG, Dougherty PM (1998). Patterns of bursting occurring in thalamic cells during parkinsonian tremor. Neuroscience.

[13] Pedrosa DJ, Quatuor E-L, Reck C, Pauls KAM, Huber CA, Visser-Vandewalle V, Timmermann L (2014). Thalamomuscular coherence in essential tremor: hen or egg in the emergence of tremor?. J Neurosci.

[14] Schnitzler A, Munks C, Butz M, Timmermann L, Gross J (2009). Synchronized brain network associated with essential tremor as revealed by magnetoencephalography. Mov Disord.

[15] Weerasinghe G, Duchet B, Cagnan H, Brown P, Bick C, Bogacz R (2019). Predicting the effects of deep brain stimulation using a reduced coupled oscillator model. PLoS Comput Biol.

[16] Gillies A, Willshaw D, Li Z (2002). Subthalamic-pallidal interactions are critical in determining normal and abnormal functioning of the basal ganglia. Proc Biol Sci.

[17] Pavlides A, Hogan SJ, Bogacz R (2015). Computational models describing possible mechanisms for generation of excessive beta oscillations in Parkinson’s disease. PLoS Comput Biol.

[18] Yousif N, Mace M, Pavese N, Borisyuk R, Nandi D, Bain P (2017). A network model of local field potential activity in essential tremor and the impact of deep brain stimulation. PLoS Comput Biol.

[19] Haidar I, Pasillas-Lepine W, Chaillet A, Panteley E, Palfi S, Senova S (2016). Closed-loop firing rate regulation of two interacting excitatory and inhibitory neural populations of the basal ganglia. Biol Cybern.

[20] Velarde OM, Mato G, Dellavale D (2017). Mechanisms for pattern specificity of deep-brain stimulation in Parkinson’s disease. PLoS ONE.

[21] Pollina B, Benardete D, Noonburg VW (2003). A periodically forced Wilson–Cowan system. SIAM J Appl Math.

[22] Winfree AT (2001). The geometry of biological time.

[23] Brown E, Moehlis J, Holmes P (2004). On the phase reduction and response dynamics of neural oscillator populations. Neural Comput.

[24] Izhikevich EM (2007). Dynamical systems in neuroscience.

[25] Guillamon A, Huguet G (2009). A computational and geometric approach to phase resetting curves and surfaces. SIAM J Appl Dyn Syst.

[26] Wedgwood KC, Lin KK, Thul R, Coombes S (2013). Phase-amplitude descriptions of neural oscillator models. J Math Neurosci.

[27] Castejón O, Guillamon A, Huguet G (2013). Phase-amplitude response functions for transient-state stimuli. J Math Neurosci.

[28] Butterworth S (1930). On the theory of filter amplifiers. Wirel Eng.

[29] Storey JD, Taylor JE, Siegmund D (2004). Strong control, conservative point estimation and simultaneous conservative consistency of false discovery rates: a unified approach. J R Stat Soc, Ser B, Stat Methodol.

[30] Benjamini Y, Krieger AM, Yekutieli D (2006). Adaptive linear step-up procedures that control the false discovery rate. Biometrika.

[31] Cagnan H, Weerasinghe G, Brown P. Tremor data measured from essential tremor patients subjected to phase-locked deep brain stimulation. Oxford. 2019. 10.5287/bodleian:xq24eN2Km. https://data.mrc.ox.ac.uk/data-set/tremor-data-measured-essential-tremor-patients-subjected-phase-locked-deep-brain.

[32] Onslow AC, Jones MW, Bogacz R (2014). A canonical circuit for generating phase-amplitude coupling. PLoS ONE.

[33] Palva JM, Palva S, Kaila K (2005). Phase synchrony among neuronal oscillations in the human cortex. J Neurosci.

[34] Yang H, Shew WL, Roy R, Plenz D (2012). Maximal variability of phase synchrony in cortical networks with neuronal avalanches. J Neurosci.

[35] Cagnan H, Duff EP, Brown P (2015). The relative phases of basal ganglia activities dynamically shape effective connectivity in Parkinson’s disease. Brain.

[36] Borisyuk RM, Kirillov AB (1992). Bifurcation analysis of a neural network model. Biol Cybern.

[37] Wilson D, Ermentrout B (2018). An operational definition of phase characterizes the transient response of perturbed limit cycle oscillators. SIAM J Appl Dyn Syst.

[38] Kralemann B, Cimponeriu L, Rosenblum M, Pikovsky A, Mrowka R (2008). Phase dynamics of coupled oscillators reconstructed from data. Phys Rev E.

[39] Oprisan SA (2017). A consistent definition of phase resetting using Hilbert transform. Int Sch Res Not.

[40] Glass L, Mackey MC (1988). From clocks to chaos: the rhythms of life.

[41] Monga B, Wilson D, Matchen T, Moehlis J (2019). Phase reduction and phase-based optimal control for biological systems: a tutorial. Biol Cybern.

[42] Wilson D, Moehlis J (2014). Optimal chaotic desynchronization for neural populations. SIAM J Appl Dyn Syst.

[43] Ott E, Antonsen TM (2008). Low dimensional behavior of large systems of globally coupled oscillators. Chaos, Interdiscip J Nonlinear Sci.

[44] Bick C, Goodfellow M, Laing CR, Martens EA. Understanding the dynamics of biological and neural oscillator networks through mean-field reductions: a review. 2019. arXiv:1902.05307. 10.1186/s13408-020-00086-9 PMC7253574 32462281

[45] Beaumont MA (2019). Approximate Bayesian computation. Annu Rev Stat Appl.

[46] Lueckmann J-M, Goncalves PJ, Bassetto G, Öcal K, Nonnenmacher M, Macke JH (2017). Flexible statistical inference for mechanistic models of neural dynamics. Advances in neural information processing systems.

[47] Chen LL, Madhavan R, Rapoport BI, Anderson WS (2013). Real-time brain oscillation detection and phase-locked stimulation using autoregressive spectral estimation and time-series forward prediction. IEEE Trans Biomed Eng.

[48] Ueta T, Chen G (2003). On synchronization and control of coupled Wilson–Cowan neural oscillators. Int J Bifurc Chaos.

[49] Raethjen J, Deuschl G (2012). The oscillating central network of essential tremor. Clin Neurophysiol.

[50] Engel AK, Gerloff C, Hilgetag CC, Nolte G (2013). Intrinsic coupling modes: multiscale interactions in ongoing brain activity. Neuron.

[51] Fries P (2015). Rhythms for cognition: communication through coherence. Neuron.

[52] Bedrosian E (1963). A product theorem for Hilbert transforms. Proc IEEE.

[53] Torczon V (1997). On the convergence of pattern search algorithms. SIAM J Optim.

[54] Audet C, Dennis JE (2003). Analysis of generalized pattern searches. SIAM J Optim.

[55] Särkkä S, Solin A (2019). Applied stochastic differential equations.

